# Sustainable RCCI engine operation with an ANN based novel tri-fuel approach

**DOI:** 10.1038/s41598-025-09984-y

**Published:** 2025-07-29

**Authors:** P. V. Elumalai, Chin-Shiuh Shieh, M. Sreenivasa Reddy, S. Rama Sree, Shashikumar Krishnan

**Affiliations:** 1 Department of Mechanical Engineering, Aditya University, Surampalem, AP India; 2 Research Institute of IoT Cybersecurity, Department of Electronic Engineering, National Kaohsiung University of Science and Technology, Kaohsiung, Taiwan; 3 Department of Electronic Engineering, National Kaohsiung University of Science and Technology, Kaohsiung, Taiwan; 4 Department of Computer Science and Engineering, Aditya University, Surampalem, Andhra Pradesh India; 5 Faculty of Artificial Intellignece and Engineering (FAIE), Multimedia University, Persiaran Multimedia, 63100 Cyberjaya, Selangor Malaysia

**Keywords:** RCCI engine, Tri-fuel approach, Pugh matrix, Sustainability assessment, Machine learning algorithms, ANN

## Abstract

This research delves into a novel tri-fuel RCCI engine strategy that uses diesel as the base fuel, biodiesel from *Andropogon narudus*, and hydrogen as a reactivity promoter to enhance combustion efficiency and enhance environmental sustainability. The tested blends, BD80H20 and BD70H30, showed a 3–5% improvement in Brake Thermal Efficiency (BTE) compared to conventional diesel at full load operation. Hydrogen-rich blends recorded a 5–8% brake-specific fuel economy improvement over diesel and B20 at both 75% and 100% engine loads. The biodiesel has made a substantial reduction in hydrocarbon (HC) and carbon monoxide (CO) emissions. Specifically, B20 recorded a 15% decrease in HC and 12% decrease in CO emission compared to straight diesel, while hydrogen blends register another decrease of 20–25% in CO emissions. The addition of hydrogen resulted in a rough estimate of 10–15% increase in the emissions of carbon dioxide (CO_2_) and nitrogen oxides (NOx), reflecting an emission trade-off. The smoke opacity decrease varied between 18 and 25% for hydrogen–biodiesel blends, reflecting an increased combustion efficiency level. The increase in in-cylinder peak pressure by 5–10% with hydrogen reflects an accelerated and efficient combustion process. A sustainability analysis by means of a Pugh matrix and Kiviat plot showed that BD80H20 was the most sustainable mixture. The ANN validation proved to be of excellent prediction quality, with RMSE values ranging from 0.9965 to 0.9996, and MPAE less than 4%.

## Introduction

The continued exploitation of fossil fuels has fueled industrial and societal advancement; however, it has done so at the expense of boosting greenhouse gas emissions. This has led to cleaner energy sources. Internal combustion engines, especially diesel engines, are under stringent emissions standards to minimize environmental degradation. Because of their efficiency in fuel and economy, single-cylinder direct injection (DI) diesel engines are best utilized in agricultural uses and small power generation^1^. Because of the nature of the design, it is difficult to make them meet the emissions standards, thus the necessity for complex combustion processes^2^. Reactivity Controlled Compression Ignition (RCCI), which is developed at the University of Wisconsin-Madison Engine Research Centre, enhances the combustion techniques^3^. Burning is highly controlled by applying a carefully selected blend of fuels with different reactivities^4^. RCCI leads to high reductions in NOx and PM emissions and an increase in fuel efficiency. RCCI meets the requirements of emissions without expensive after-treatment systems as compared to diesel engines^5^. The disadvantages are high hydrocarbon (HC) and carbon monoxide (CO) emissions at low loads, as well as high pressure rise rates at high loads, which require thorough optimization over a wide range of operating conditions^6^. To improve the environmental friendliness of internal combustion engines, it is necessary to research alternative fuels^7^. Biodiesel has been widely researched for its potential to reduce HC, CO, and smoke emissions. The oxygen content results in elevated NOx emissions^8^. Experiments have been performed on ethanol and water-emulsified biodiesel to remedy this situation^9^. The high burning rate of hydrogen, as well as its wide flammability range, greatly enhances combustion efficiency with reduced soot production^10^. Like biodiesel, it increases NOx emissions at high engine loads, which requires careful combustion control^11^. *Andropogon narudus* biofuel is a source of clean energy that can help decrease carbon emissions substantially^12^. Research on its diesel blends in compression ignition engines showed enhanced brake thermal efficiency and emissions. Sustainability trials based on the Pugh matrix show it as a promising alternative fuel source^13,14^. The use of biodiesel, hydrogen, and *A. narudus* biofuel within a tri-fuel system provides a way of increasing combustion efficiency while lessening environmental impacts^15^. A tri-fuel RCCI engine system employs diesel, hydrogen, and *Andropogon narudus* biofuel to achieve maximum efficiency and minimize emissions^16,17^.

Combustion begins with diesel, accompanied by *Andropogan narudus* biofuel as a carbon–neutral renewable fuel^18^. Hydrogen, noted for its reduced reactivity, enables flame spread and lean combustion support; yet it is not used as a pilot or primary fuel individually^19^. Research from past investigations into diesel-hydrogen RCCI engines suggests improvement in combustion efficiency; yet, higher levels of hydrogen cause increased NOx emissions^20,21^.

The feature of RCCI to adjust the reactivity of fuel by utilizing sophisticated injection strategies offers a convincing method of managing emissions. Even though tri-fuel RCCI systems show promising aspects, extensive studies are needed to confirm and continue their development^22,23^. It is important to increase the performance of tri-fuel RCCI engines and reduce pollution in real-world applications^24^. Artificial Neural Networks (ANNs) prove high effectiveness in achieving this^13^. Artificial neural networks can identify subtle relations between injection timing, fuel ratio, intake air temperature, and their implications on performance and emissions^25^. Forecasting reduces the necessity of experimental trials and facilitates the design of more efficient engine control systems^26^. The use of genetic algorithms with artificial neural networks enables the identification of engine configurations that maximize fuel efficiency and reduce emissions. Artificial neural networks can optimize tri-fuel RCCI engines with dynamic fuel blends, since they learn by experimenting^27^. The combination of machine learning with RCCI combustion technologies may result in the development of sustainable and high-performance engine running^28^. In this research work, a tri-fuel RCCI engine has been studied with diesel, hydrogen, and *Andropogon narudus* biofuel as fuels^29^. A single cylinder DI diesel engine modified to RCCI mode is used in this research work^30^. The research starts with fuel blending and characterization. The respective proportions of diesel, hydrogen, and *A. narudus* biofuel will be mixed to determine calorific value, viscosity, and combustion behavior^31,32^. The properties enable fuel injection methods and provide stable RCCI operation. The next phase is engine experimentation. During RCCI operation, a single-cylinder engine will employ double-fuel injection technology^33^. The research will evaluate the impact of the tri-fuel system on several parameters such as engine response, cylinder pressure, fuel injection timing, brake thermal efficiency, and exhaust emissions like NOx, PM, CO, and HC. Machine learning methods will enhance optimization^34^.

Artificial neural networks will be trained using experimental data and will be able to predict engine performance under different conditions. Optimization algorithms will calculate injection timing, fuel–air ratio, and other parameters to produce maximum efficiency and emissions to the lowest levels. This analysis methodology is set to significantly enhance combustion stability and maximize engine efficiency. The Pugh matrix will be utilized to evaluate end of life sustainability. The specified decision-making tool will evaluate emissions, fuel economy, cost, resource availability, and infrastructure requirements of the tri-fuel RCCI system versus diesel engines. The Pugh matrix analyzes the criteria for the projected system’s economic and environmental feasibility. A tri-fuel engine with diesel, hydrogen, and *Andropogon narudus* biofuel, built around RCCI principles of combustion, augmented by machine learning-based real-time optimization and sustainability analysis with the Pugh matrix, has the capability to revolutionize the cleanliness of internal combustion engines^35^. This research investigates leading-edge combustion technology, low-carbon fuels, and advanced control systems as potential options to diesel engines. Despite the difficulties presented by fuel storage, infrastructure, and control complexity, ongoing exploration and development in the area present encouraging directions for future sustainable mobility and power generation solutions. This research employs novel experimental techniques and AI optimization to create sustainable technologies for internal combustion engines.

## Materials and methods

### Fuel biography

#### *Andropogan narudus* (AN)

The Citronella Grass, scientifically known as *Andropogon narudus*, is a perennial species of grass which is native to the tropical as well as subtropical regions. This plant is valued for its aromatic essential oil, which is widely used for the manufacture of perfumes and insect repellents. In addition to the traditional use, *A. nardus* is now being considered as a source of feedstock for biofuels due to its high biomass and its ease in growing on marginal lands. Its high concentration of cellulose with high fiber content makes it potential for various conversion technologies that could be useful in biofuels production. Although this holds great promise as a source of renewable energy, there are still many challenges to perfect the cultivation and processing technology to make low-cost biofuels feasible^15,34^.

##### FTIR and GCMS analysis of *Andropogan narudus* fuel

Fourier-transform: Infrared spectroscopy (FTIR) is yet another often used analytical technique comparing infrared intensity versus wavelength (or wavenumber) of light. Determining chemical bond vibrations in functional groups provides important understanding of the molecular structure and content of a sample. Every type of bond has a different spectral fingerprint to be used and vibrates at a designated frequency. This allows one to track chemical reactions, identify specific molecules, or establish material properties.

**Fig. 1:**
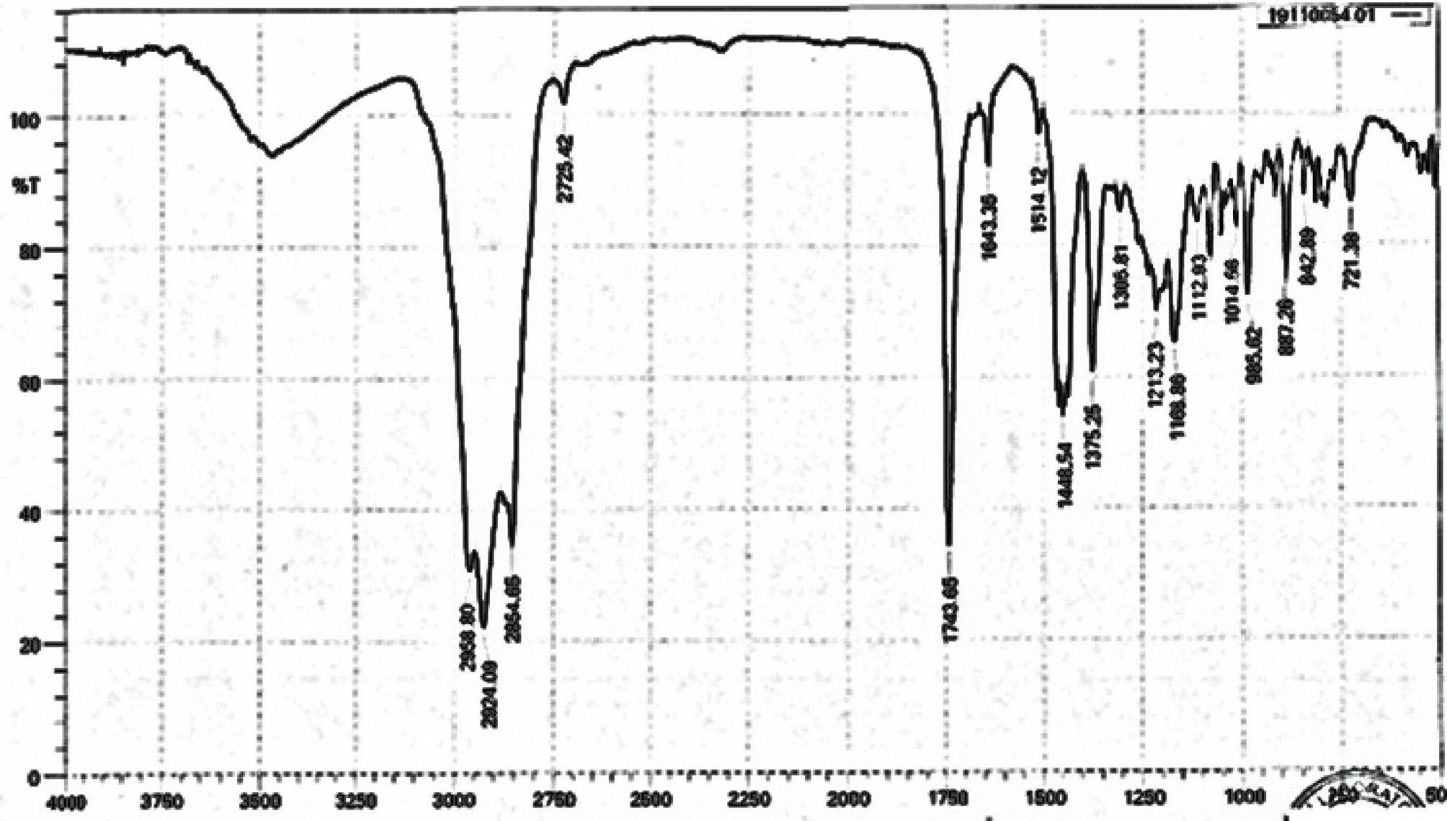
Represents the Fourier transform infrared spectroscopy of the *Andropogan narudus* fuel.

The several absorption peaks shown in this Fig. 1 demonstrate the existence of several functional groups in the FTIR spectrum. Consistent with aliphatic –CH_2_-groups, C–H stretching vibrations explain the strong peaks seen at 2959.89, 2901.03, and 2866.66 cm⁻^1^. Commonly with ester, ketone, or aldehyde functional groups, a sharp peak at 2726.42 cm⁻^1^ denotes an aldehydic C–H stretch; a sharp absorption at 1734.08 cm⁻^1^ shows a carbonyl (C=O) stretching vibration. Other bands at 1643.98 cm⁻^1^ and 1514.12 cm⁻^1^ could indicate C=C stretching or aromatic ring vibrations; the peak at 1446.54 cm⁻^1^ shows C–H bending vibrations. Surely the 1375.25 cm⁻^1^ absorbance results from bending of methyl groups. Furthermore, indicated by the complex pattern in the 1230–1020 cm⁻^1^ area with maxima at 1230.23, 1186.89, 1101.49, and 1022.20 cm⁻^1^ are C–O stretching vibrations typical for esters or ethers. Finally, the 720.38 cm⁻^1^ absorption is caused by characteristic for a straight alkyl chain methylene rocking mode of long chains. These spectroscopic features point to the sample might include synthetic polymers like polyesters, plasticizers, or ester-based compounds—perhaps long-chain fatty acid esters—or both.

**Fig. 2:**
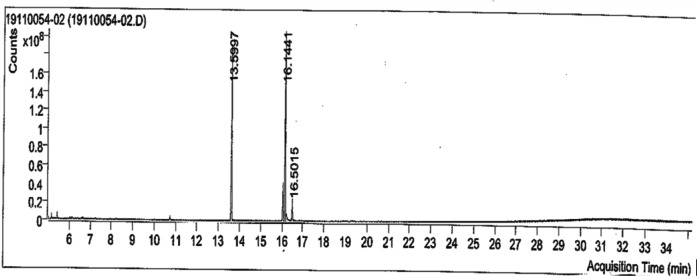
GCMS analysis of *Andropogan narudus*.

Figure 2 shows the analysis using GC–MS revealed the presence of various fatty acid methyl esters (FAMEs), which suggests the composition of natural fats or biodiesel products. Four significant compounds were identified through the examination of their peak regions, spectral match scores, and retention times. These compounds were derivatives of fatty acids, both saturated and unsaturated. At a retention time of 13.5997 min, methyl palmitate was identified, suggesting the presence of an ester derived from a saturated C16 fatty acid. Two isomeric compounds—both unsaturated C18 esters—were identified at 16.1441 min: methyl ester and trans-13-octadecenoic acid, Z-; 9-octadecenoic acid, Z- At 16.5015 min, another unsaturated C18 ester, methyl stearate, was identified. The elevated match scores and significant peak areas of these compounds confirm their primary role in the overall composition of the sample. Table 1 shows the findings are presented in the table below:

**Table 1:** The compounds identified from GCMS analysis.

Retention time (min)	Compound name	Formula	CAS number	Match score	Peak area
13.5997	Methyl palmitate	C_17_H_34_O_2_	112-39-0	84.7	362,237,560
16.1441	9-Octadecenoic acid (Z)-, methyl ester	C_19_H_36_O_2_	112-62-9	85.5	318,675,544
16.1441	Trans-13-octadecenoic acid, methyl ester	C_19_H_36_O_2_	1000333-61-3	83.2	317,355,357
16.5015	Methyl stearate	C_19_H_38_O_2_	112–61-8	81.1	43,786,272

#### Hydrogen (H_2_)

Hydrogen has been considered one of the elements with the most significant potential, being identified as the most abundant element in the universe, especially in its energy density and in its combustion characteristics that give way to low emission levels. Material sources for hydrogen can come from fossil as well as renewable energies. But it depends on the availability of low-cost and sustainable production methods, particularly green hydrogen, produced through renewable electrolysis. The major bottlenecks remain in storage, transportation, and infrastructure building that limit general usage of these technologies. Research now focuses on cost reduction for the production, improved storage techniques, and enhanced efficiency of fuel cells. This lays the basis through which hydrogen could become an important part of attaining a renewable energy future for transportation, electricity generation, and industrial use^36^.

Hydrogen is very reactive and hence considered as a promising test fuel to integrate into RCCI engines for precise control over combustion, which brings with it an opportunity for higher efficiency and fewer emissions. With proper hydrogen injection strategies to inject small pulses of hydrogen, the combustion of the less reactive primary fuel, such as diesel or biofuel can be accurately controlled. This kind of controlled combustion in RCCI mode, besides reducing NOx emissions, also helps to reduce particulate matter, hydrocarbon, and carbon monoxide with the clean-burning nature of hydrogen. The challenges are hydrogen storage and delivery issues, the need for sophisticated control systems in order to manage the pilot injection, and engine durability when using hydrogen^30,37^.

In the current study, a hydrogen (H_2_) flow rate of 4 L per minute (LPM) was chosen due to its critical role in both the reaction kinetics and overall process efficiency. Hydrogen is an important reducing agent or carrier gas in many catalytic and thermal processes, especially in hydrogenation and pyrolysis routes. A flow rate of 4 LPM provides an optimal balance between preserving a reducing environment and preventing excessive dilution of reactive intermediates. Lower flow rates can lead to inadequate hydrogen availability, which may cause incomplete reactions, the production of unwanted by-products, or catalyst deactivation through carbon deposition. In contrast, extremely high flow rates would reduce residence time, influencing the rate of conversion and efficiency in the system as well as increasing gas usage. The optimum rate selected here as 4 LPM is also in consonance with values demonstrated in similar studies, wherein the efficiency and product selectivity during reaction were achieved under conditions of moderate hydrogen flow rates. Further, initial trials further confirmed that stable chemical and thermal conditions were introduced with this rate of flow, so that comparable and reproducible results were seen through repeated operations. Therefore, the rate of 4 LPM was finalized as an operational parameter for optimal study, between maximizing reaction yield and conserving resources^36,38^.

In this study, hydrogen was supplied to the engine through a port fuel injection (PFI) system to improve reactivity and combustion properties under RCCI operation. Hydrogen was fed from a high-pressure cylinder and went through a two-stage regulation system for safe and accurate control. First, the pressure was brought down from around 150 bar to about 4 bar with a primary regulator and then by a secondary regulator which provided a stable working pressure of about 2 bar at the injector. The flow rate of hydrogen was adjusted to 4 L per minute (LPM) after initial trials were conducted to arrive at the optimum combustion without knocking or excessive formation of NOx. A mass flow controller Alicat Scientific was utilized to supply stable flow during engine run. During the intake stroke, hydrogen was supplied to the intake port through a solenoid injector synchronized with the engine’s ECU to provide optimal air–fuel mixing before compression. The setup consisted of flame arrestors and non-return valves to ensure safe operation throughout the hydrogen supply line. This setup enabled efficient use of hydrogen while providing safe engine operation.

#### Fuel nomenclature

Table 2 presents an in-depth view of the nomenclature used in fuel within this study, paying particular attention to a tri-fuel system. The biofuel used in this research is *Andropogon narudus*, which has been mixed with diesel at 20:80 to become AN20 + Diesel, a fuel to be used in this research. The main fuel is herein termed BD and is introduced in different percentages, between 90 and 70%, while hydrogen is added as a pilot fuel and the percentages rise from 10 to 30% correspondingly^18^. These percentages express the contribution each type of fuel has towards the total energy input derived from fuels. The hydrogen flow rate is maintained at a constant 4 L per minute for all fuel combinations tested, thus allowing the researchers to explore the effect of different hydrogen pilot injection on the combustion dynamics inside the engine, likely an RCCI engine. The BD90H10 is the designation for a formulation that consists of 90% AN20 mixed with Diesel and 10% hydrogen, which is dosed at a rate of 4 L per minute^39^.

**Table 2:** Fuel nomenclature.

Nomenclature of the fuel used			
Biofuel	*Andropogan narudus*		Flow of hydrogen
B20 + Diesel	AN20 + Diesel		
Injection	Primary injection	Pilot injection	LPM
BD90H10	90(AN20 + Diesel)	10% (Hydrogen)	4
BD85H15	85(AN20 + Diesel)	15% (Hydrogen)	4
BD80H20	80(AN20 + Diesel)	20% (Hydrogen)	4
BD75H25	75(AN20 + Diesel)	25% (Hydrogen)	4
BD70H30	70(AN20 + Diesel)	30% (Hydrogen)	4

Fuel properties^34,36^:

Table 3 illustrates a comparison of basic fuel properties for diesel, Andropogon nardus (AN) biofuel, and hydrogen (H_2_). In this regard, diesel has the highest calorific value with 44.52 MJ/kg, followed by ammonium nitrate at 36.27 MJ/kg. On the other hand, hydrogen exhibits a much higher calorific value of 142.18 MJ/kg, meaning it has much more energy per unit mass. Diesel shows the highest kinematic viscosity of 3.9 cSt, meaning that it has more resistance to flow than AN with a kinematic viscosity of 4.18 cSt, and hydrogen, which has a kinematic viscosity of 0.0875 cSt. Ammonium nitrate (AN) is the densest at 910 kg/m^3^. Diesel fuel, second most dense, is at 820 kg/m^3^. Hydrogen of course shows the least density of 0.08376 kg/m^3^. Diesel had the highest flash point among the given materials, at 76 °C and is thus least flammable compared to the three. *Andropogan narudus* is the least dense. Among the materials compared, it possesses a lower flash point of 50 °C. Besides that, the flash point for hydrogen is NA because of its gaseous state. AN requires the highest energy input in vaporization at 305 kJ/kg. It is followed by diesel fuel that, at 262 kJ/kg, is the second highest in need for energy input in vaporization. Hydrogen, on the other hand, has a significantly lower energy requirement for vaporization at 14.89 kJ/kg. Thus, in conclusion, diesel has a marginally higher cetane number of 47 than AN does with its cetane number of 45. It says that there is better quality for compression ignition by diesel. On the other hand, the cetane number cannot be ascribed to hydrogen since it is not applicable. The differences observed highlight the dissimilar characteristics of each fuel type and, thus, their repercussions on engine operation and manipulation^40^.

**Table 3:** Fuel properties of the fuel used in the investigation.

Properties	Units	ASTM Standards	Diesel	AN	H_2_
Calorific value	MJ/kg	ASTM D 5865	44.52	36.27	142.18
Kinematic viscosity, 40 °C	cSt	ASTM D 445	3.9	4.18	0.0875
Density	kg/m^3^	ASTM D 4052	820	910	0.08376
Flash point	°C	ASTM D 92	76	50	NA
Latent heat of vaporization	kJ/kg	ASTM E 2071	262	305	14.89
Cetane number		ASTM D 613	47	45	NA

### Engine set up

**Fig. 3:**
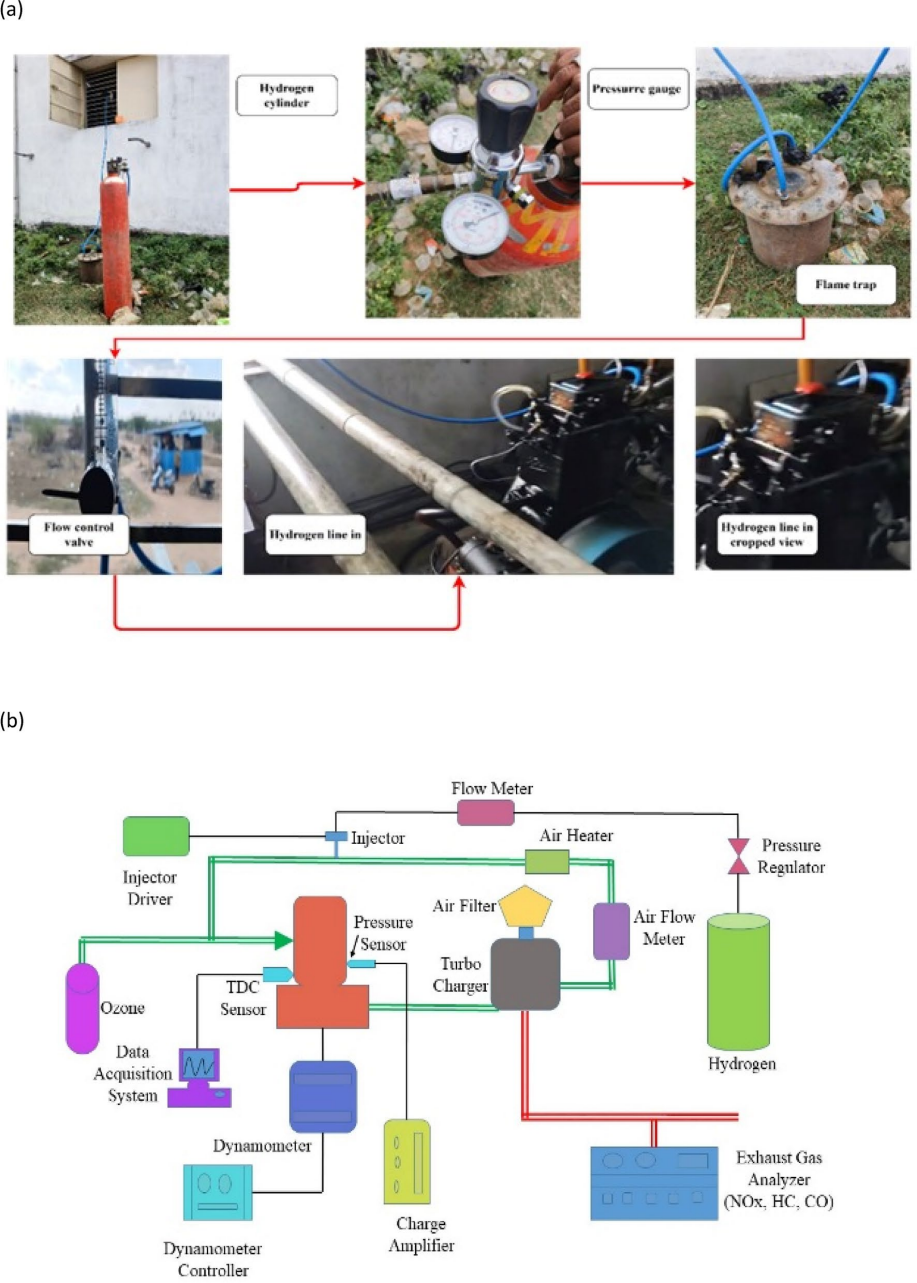
(**a**) Actual engine set up. (**b**) Schematic engine set up.

Figure 3a actual experimental setup used for testing an RCCI engine and Fig. 3b schematic Engine Setup. This engine, originally a CI engine, was modified to incorporate a manifold controlled by an electronic control unit and an injection system. A vaporizer unit was equipped with an air heater. During operation, hydrogen was introduced into the inlet manifold, while the remaining fuel was directly injected into the cylinder using a direct injection system. The experiment tested with hydrogen as pilot in various proportions of 10%, 15%, 20%,25% and 30% and BD fuel in 90%, 85%, 80%,75% and 70%. The fuel flow of primary injection is restricted using a flow control valve to ensure hydrogen is injected as pilot fuel in respective volume ratios after calculating the flow of primary fuel without hydrogen initially. To avoid fuel condensation on the cylinder walls, biodiesel was preheated and effectively vaporized at a controlled temperature of 205 °C. Tests were performed at 0 to 100% engine load to optimize combustion phasing using reactivity control strategies. An AVL Taigas analyzer was employed to measure exhaust emissions, and an AVL smoke meter was used to assess smoke opacity. In-cylinder pressure was monitored and recorded using a piezoelectric pressure sensor in conjunction with a crank angle encoder. An AVL-INDI MICRA DAQ system, connected to the engine, facilitated data acquisition and combustion analysis. Engine performance metrics, including power, speed, torque, fuel consumption, and lubrication consumption, were precisely analyzed using an Eddy dynamometer and a dedicated controller^26^.

#### Uncertainty analysis

In the process of continuous experimental studies, it has been found that errors may come up due to fluctuations in environmental conditions and variations in the conduct of experimental protocols. Uncertainty analysis forms an important methodology for reducing the effects of probable errors. Although there is likely to be expert opinion that can be derived on the extent of errors made, it must be realized that there also exists a danger of bias from these estimates. To ensure that these complications are overcome, and the resultant data is quite reliable, numerous readings and measurements are taken from the continuous working of the engine. The repeated data gathering enables the system’s behavior to be understood more deeply and quantifies uncertainty associated with the measured parameters. In addition, through the examination of the distribution of these different measurements, researchers can enhance the estimation of the true value of each parameter and the range within which it is likely to reside. This methodology proves to make the experimental findings more robust and allows the derivation of more substantiated conclusions. Table 3 shows the values of uncertainty of the parameters measured^41^.

$$\begin{aligned} {\text{Total}}\,{\text{ uncertainty}} & = \sqrt {\left( {U_{{{\text{BTE}}}} } \right)^{{2}} + \, \left( {U_{{{\text{BSFC}}}} } \right)^{{2}} + \, \left( {U_{{{\text{UBHC}}}} } \right)^{{2}} + \, \left( {U_{{{\text{NOx}}}} } \right)^{{2}} + \, \left( {U_{{{\text{CO}}}} } \right)^{{2}} + \, \left( {U_{{{\text{Smoke}}}} } \right)^{{2}} } \\ & = \sqrt {\left( {U_{{{\text{BTE}}}} } \right)^{{2}} + \, \left( {U_{{{\text{BSFC}}}} } \right)^{{2}} + \, \left( {U_{{{\text{UBHC}}}} } \right)^{{2}} + \, \left( {U_{{{\text{NOx}}}} } \right)^{{2}} + \, \left( {U_{{{\text{CO}}}} } \right)^{{2}} + \, \left( {U_{{{\text{Smoke}}}} } \right)^{{2}} } \\ & = { 1}.{93}\% \\ \end{aligned}$$

The Table 4 delineates an uncertainty analysis concerning critical engine performance and emission parameters. The Brake Thermal Efficiency (BTE) and smoke emissions demonstrate the greatest degree of uncertainty, quantified at 0.9%, indicating that these measurements possess the lowest level of precision. The Brake Specific Fuel Consumption (BSFC) and Carbon Monoxide (CO) exhibit a marginally reduced uncertainty of 0.8%, whereas the uncertainty associated with Hydrocarbon (HC) emissions is recorded at 0.7%. Nitrogen Oxides (NOx) exhibit the lowest level of uncertainty at 0.6%, signifying the highest reliability in measurement among the parameters evaluated. This analysis elucidates the diverse degrees of confidence linked to the measurement or calculation of each parameter, which is essential for the interpretation of results and the formulation of informed decisions.

**Table 4:** Mentions the uncertainty of the parameters measured.

Parameters	Uncertainty	Measuring instruments used in the Experimentation	Uncertainties (%)
BTE	0.9	–	± 0.9
BSFC	0.8	Flow meter	± 0.8
HC	0.7	AVL exhaust gas analyzer	± 0.7
CO	0.8	AVL exhaust gas analyzer	± 0.8
NOx	0.6	AVL exhaust gas analyzer	± 0.6
Smoke	0.9	AVL DI	± 0.9

### ANN model

Artificial Neural Networks (ANN) are computational frameworks inspired by the neural architecture of the human brain. These models, composed of interconnected units or neurons, work collaboratively to identify patterns, learn from data, and make predictions based on acquired knowledge. Among their diverse applications, ANN models have been widely employed to predict performance metrics and emissions in diesel engines operating on biodiesel blends. By training these models with experimental data—such as engine speed, load conditions, and fuel properties—they can estimate parameters like brake-specific fuel consumption, brake thermal efficiency, exhaust gas temperature, and emissions of NOx, CO, and particulate matter.

For experimental setups involving hydrogen as a low-reactivity fuel (LRF), along with diesel and duckweed bio-oil as high-reactivity fuels (HRF), ANN models serve as powerful tools to optimize engine performance and minimize emissions. This approach enhances the sustainability of biodiesel as an alternative energy source, addressing environmental challenges while reducing dependence on fossil fuels.

ANN models offer multiple advantages in engine research. They deliver precise predictions when provided with comprehensive datasets, enabling effective engine optimization. Adaptable and capable of learning from new data, they are versatile tools for a wide range of engine technology applications. Their ability to handle non-linear relationships makes them particularly effective in capturing the dynamic nature of engine performance. Furthermore, ANN models can integrate various learning algorithms, such as Levenberg–Marquardt (LM), Scaled Conjugate Gradient (SCG), Resilient Propagation (RP), and Broyden–Fletcher–Goldfarb–Shanno (BFGS), which are selected based on key performance metrics like the Coefficient of Determination (R), Mean Absolute Percentage Error (MAPE), and Mean Squared Error (MSE). For example, higher R values coupled with lower MSE and MAPE values indicate more accurate and reliable models.

Additionally, ANN models demonstrate robustness against incomplete or noisy data, ensuring reliability for real-world scenarios. Their rapid processing capabilities allow for real-time performance adjustments and enhancements. Scalable by design, ANN models accommodate more complex datasets and architectures over time, aligning with advancements in diesel engine technologies.

### Development of PUGH matrix

**Fig. 4:**
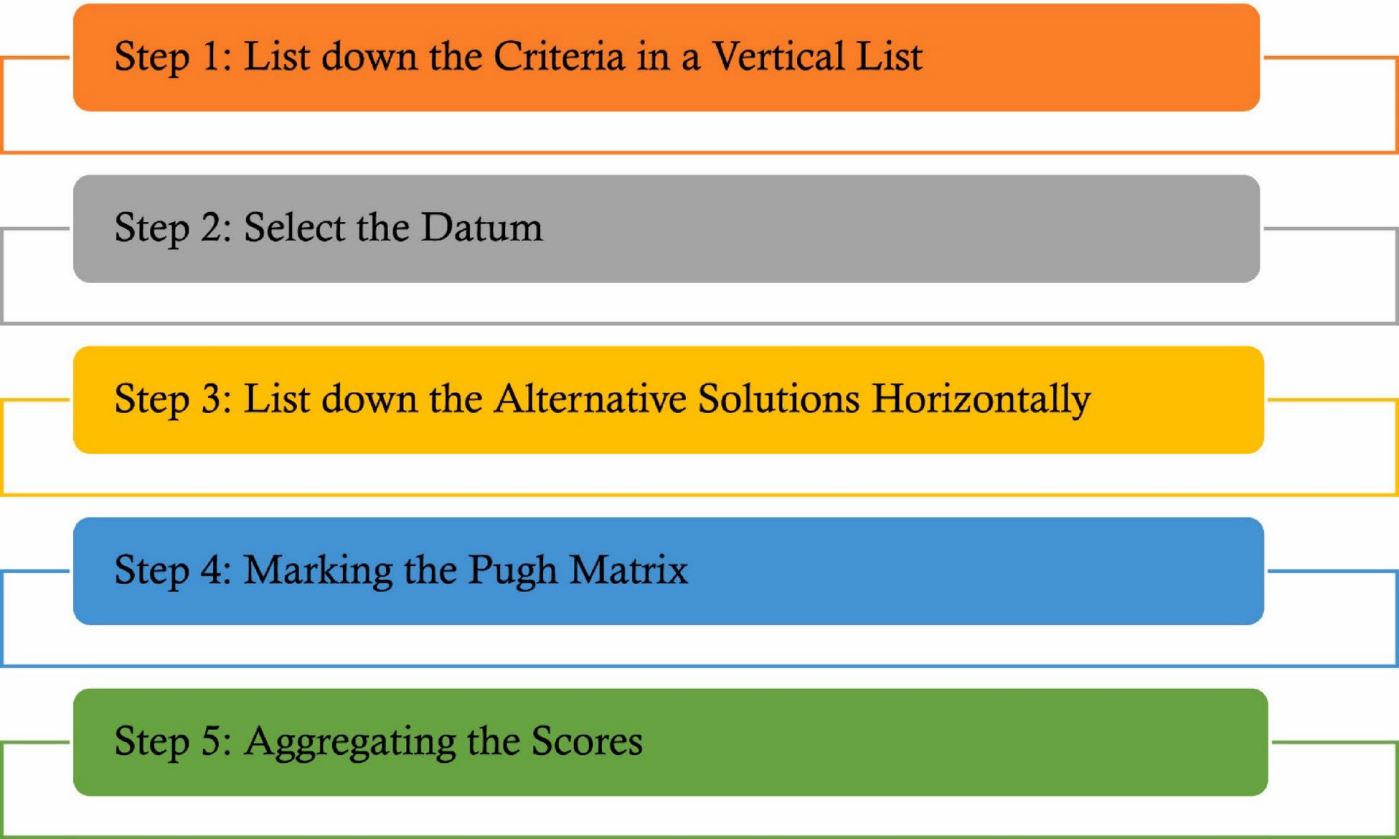
Procedures in development of PUGH matrix^31^.

The formation of a Pugh matrix is a structured approach to decision making, highly helpful when there are numerous alternatives to be weighed versus a set of defined criteria. First, a team defines the key relevant criteria to a decision-making process, ensuring that such criteria are specific and quantifiable. Then, possible options or solutions are systematically determined and enumerated. One alternative is designated as the baseline, which may be the status quo or the standard solution. Then, each of the remaining alternatives is compared to this baseline on each criterion, using a simple scoring system, for example, superior, inferior, or equal. The aggregation of scores assigned to each alternative in the evaluation process will lead to the identification of the best alternative based on the highest frequency of “better’ scores or the greatest cumulative score, if a weighted scoring system is in place. This process will enable objective comparison, encourage discussion, and help in the selection of the strongest candidates while at the same time pointing out areas that need improvement^34^. Figure 2 shows the procedures in development of PUGH matrix and Fig. 4 shows the procedures in development of PUGH matrix.

## Results and discussion

### Performance parameters

#### Brake Thermal efficiency

**Fig. 5:**
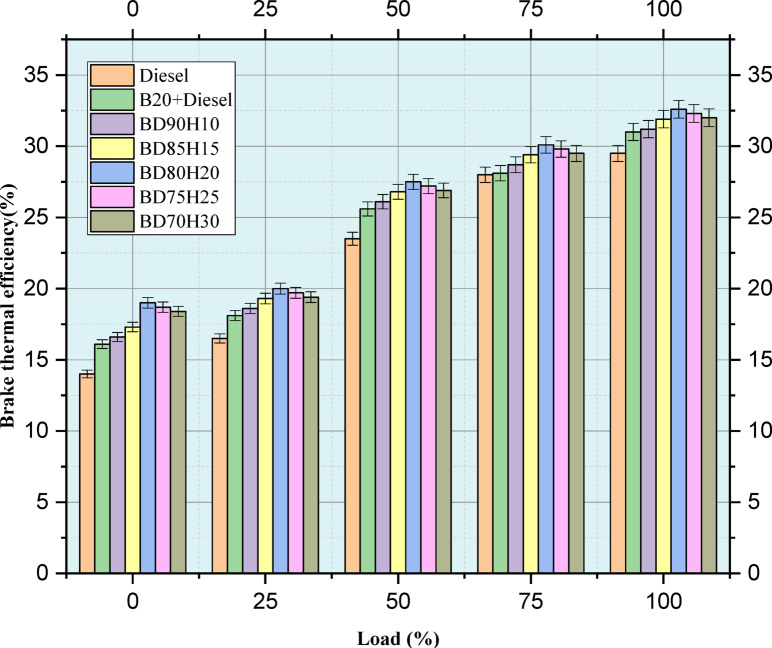
Load (%) versus BTE (%).

Figure 5 indicates the percentage of brake thermal efficiency derived from the investigation. Brake Thermal Efficiency (BTE), that is the degree to which fuel energy is converted into useful work, has been studied for various engine load conditions (0–100%) with different blends of fuels. The study has been done on a basic diesel, 20% biodiesel and 80% diesel (B20), and several BD90H10, BD85H15, BD80H20, BD75H25, and BD70H30 biodiesel-hydrogen combinations, where BD stands for biodiesel-diesel and H means that hydrogen injection was used. As expected, BTE typically increases with load for all fuel types, thus reflecting more efficient engine operation when the power is increased. Pure diesel sets the performance reference point. The B20 blend has BTE that runs slightly lower compared to pure diesel for all tested loads, with a likely causal effect being related to the decreased energy density due to biodiesel. A small amount of hydrogen in the biodiesel has a potential. Notably, these two fuels; BD80H20 and BD70H30, exhibit highest BTE values at 100% load level, 32.6 and 32 percent, respectively even higher than diesel fuel (29.5%), which implies a faster flame speed due to addition of hydrogen improving combustion characteristics hence efficiency. Of course, optimization of hydrogen proportions is critical for all hydrogen-based blends to become better than their diesel counterpart. For example, in addition to being improved over diesel at 100% load, BD85H15 (31.9%) and BD75H25 (32.3%) are at best marginal improvements. Under lighter loads the addition of hydrogen produces much lesser gains, with some blends falling even lower in BTE relative to diesel. This can be attributed by changed combustion timing or higher losses with hydrogen addition. The data suggests that careful optimization of the hydrogen fraction in biodiesel blends leads to enhanced BTE, although the effect is load-dependent and requires further investigation to fully understand the underlying mechanisms^42^.

#### Brake specific fuel consumption

**Fig. 6:**
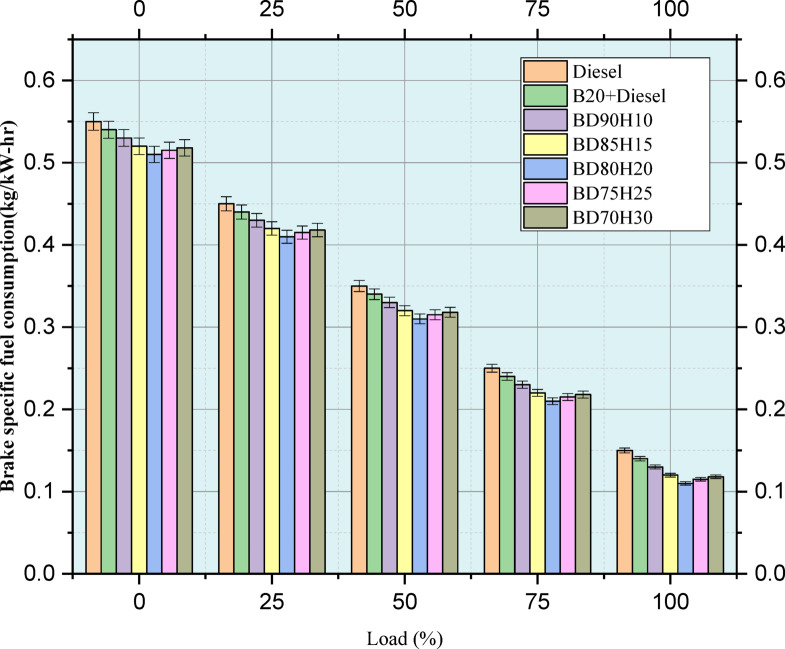
Load versus BSFC (kg/kW-h).

Figure 6 indicates fuel consumption values in the investigation. Brake specific fuel consumption (BSFC) is examined at various loads from 0 to 100% for three different fuel blends. The lowest BSFC indicates the best fuel economy. Three fuels are evaluated: standard diesel, a blend of 20% biodiesel and 80% diesel, B20, and several different blends of biodiesel and hydrogen, BD90H10, BD85H15, BD80H20, BD75H25, and BD70H30. It is generally true that BSFC increases with a higher engine load, reflecting better combustion efficiency for higher power outputs. Pure diesel is used as the baseline. The B20 blend has a slightly higher BSFC than diesel at all loads; this shows less fuel economy. The introduction of hydrogen to the biodiesel blend indicates that there can be improvements with the fuel economy. At higher loadings-particularly 75% and 100%-several hydrogen blends-hydrogen shows the lowest BSFC, both surpassing diesel and B20 at such loads. Taking the example at 100%, the loading BSFC of diesel is 0.15 kg/kW-h; meantime, BD80H20 and BD85H15 have obtained a BSFC of 0.13 kg/kW-h. The improvement has been due to hydrogen’s unique combustion properties, making the burning process completer and more efficient. Nevertheless, the right mix of hydrogen is essential. Although BD70H30 again reaches 0.13 kg/kW-h at 100% load, other blends like BD75H25 and BD90H10 only reach parity with diesel at that load. At low loads, differences in BSFC between the fuels are much reduced, and, in some instances, the blends of hydrogen display similar or marginally greater BSFC than the diesel. This load-dependent behavior indicates that hydrogen addition benefits are complex and must be further studied to optimize the fuel efficiency gain across the whole engine operating range^7^.

### Emission parameters

#### Unburned hydrocarbon

**Fig. 7:**
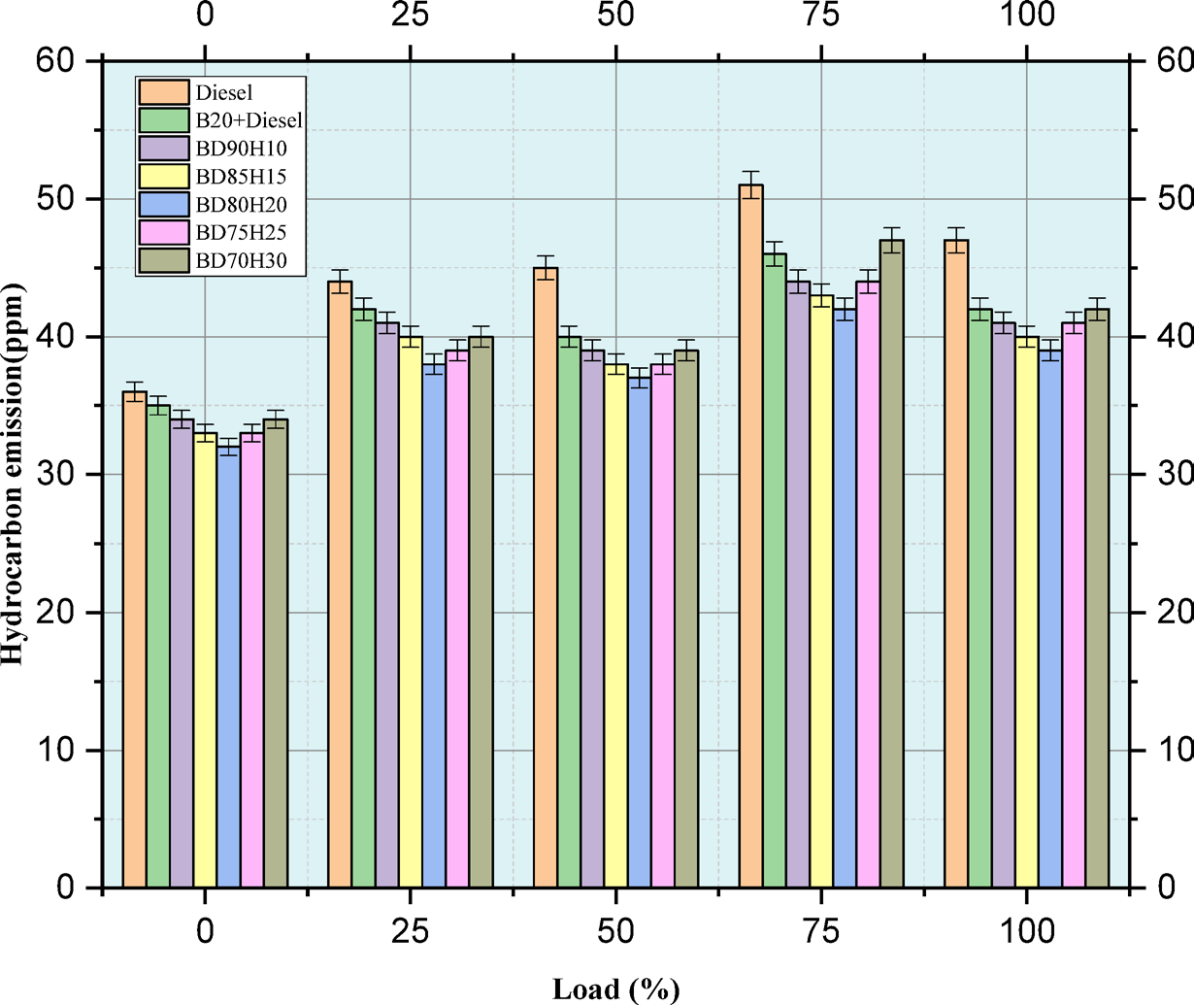
Load versus HC emission in ppm.

Figure 7 indicates the emission value of hydrocarbon in ppm. Hydrocarbon (HC) emissions, a major air pollutant, are studied at different engine loads (0–100%) for various fuel blends. Lower HC emissions are preferred as it implies that the combustion process is more complete. The present study compares standard diesel, a 20% biodiesel and 80% diesel blend (B20), and several biodiesel-hydrogen combinations (BD90H10, BD85H15, BD80H20, BD75H25, and BD70H30). Generally, the HCs emission is inversely proportional to engine load because at higher temperatures and pressures, there is more complete combustion. Pure diesel is taken as the baseline. The B20 blend shows a lower level of HCs emissions compared with pure diesel; it may be because of oxygen present in the biodiesel that increases the completeness of combustion^43^. Introduction of hydrogen in the biodiesel blend results in a mixed effect on the HCs emission. At some loads, certain hydrogen blends, such as BD80H20 at 25% load or BD70H30 at 75% load, exhibit the lowest HC emissions. This may indicate that hydrogen’s effect on combustion chemistry tends to enhance more complete oxidation of hydrocarbons. However, other hydrogen blends have similar or even slightly higher HC emissions than B20 or diesel, especially at lower loads. This may be attributed to influences such as hydrogen-induced changes in flame propagation and quenching. The overall data suggests that the effect of hydrogen addition is very sensitive both to the amount of hydrogen used and to engine load. Therefore, further experimentation is required for the optimization of the hydrogen fraction to minimize emissions of HC during the entire range of engine operating conditions^44^.

#### Carbon monoxide

**Fig. 8:**
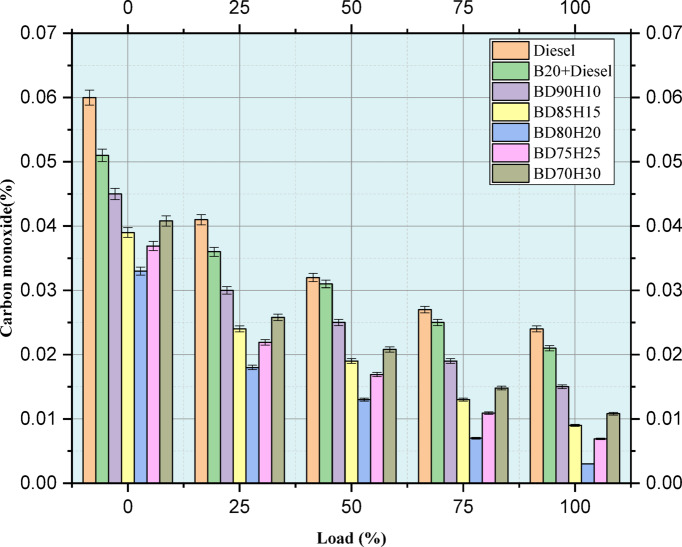
load versus CO emission in %.

Figure 8 indicates the emission value of CO emission in percentage. The CO emissions, a harmful air pollutant, are evaluated at different engine loads (0–100%) for various fuel blends. Lower CO emissions are desirable as they indicate more complete combustion. The study compares standard diesel, a 20% biodiesel and 80% diesel blend (B20), and several biodiesel-hydrogen combinations (BD90H10, BD85H15, BD80H20, BD75H25, and BD70H30). In general, CO emissions typically decrease with an increase in load due to greater temperatures and pressures promoting higher degrees of combustion completeness. Pure diesel is used as a baseline. B20, on average, shows a reduced emission of CO compared to pure diesel. It can be deduced that oxygen within the biodiesel leads to greater completeness in combustion. Introducing hydrogen in the biodiesel blend causes drastic reductions in CO emissions^45^. The hydrogen blends exhibit a much lower level of CO emissions compared to both diesel and B20 for all loads. This is very significant at high loads where CO emissions are greatly reduced. For example, at 100% load, the amount of CO emitted by diesel is 0.024 units while the range of the hydrogen blends is from 0.003 to 0.011 units. This suggests that the distinctive combustion properties of hydrogen, including high flame speed and wide flammability limits, enhance significantly the completeness of combustion, which, in turn, results in a significant decrease in CO formation. The data also indicates that hydrogen addition to biodiesel blends is an excellent strategy for reducing CO emissions throughout the engine operating range^46^.

#### Carbon dioxide

**Fig. 9:**
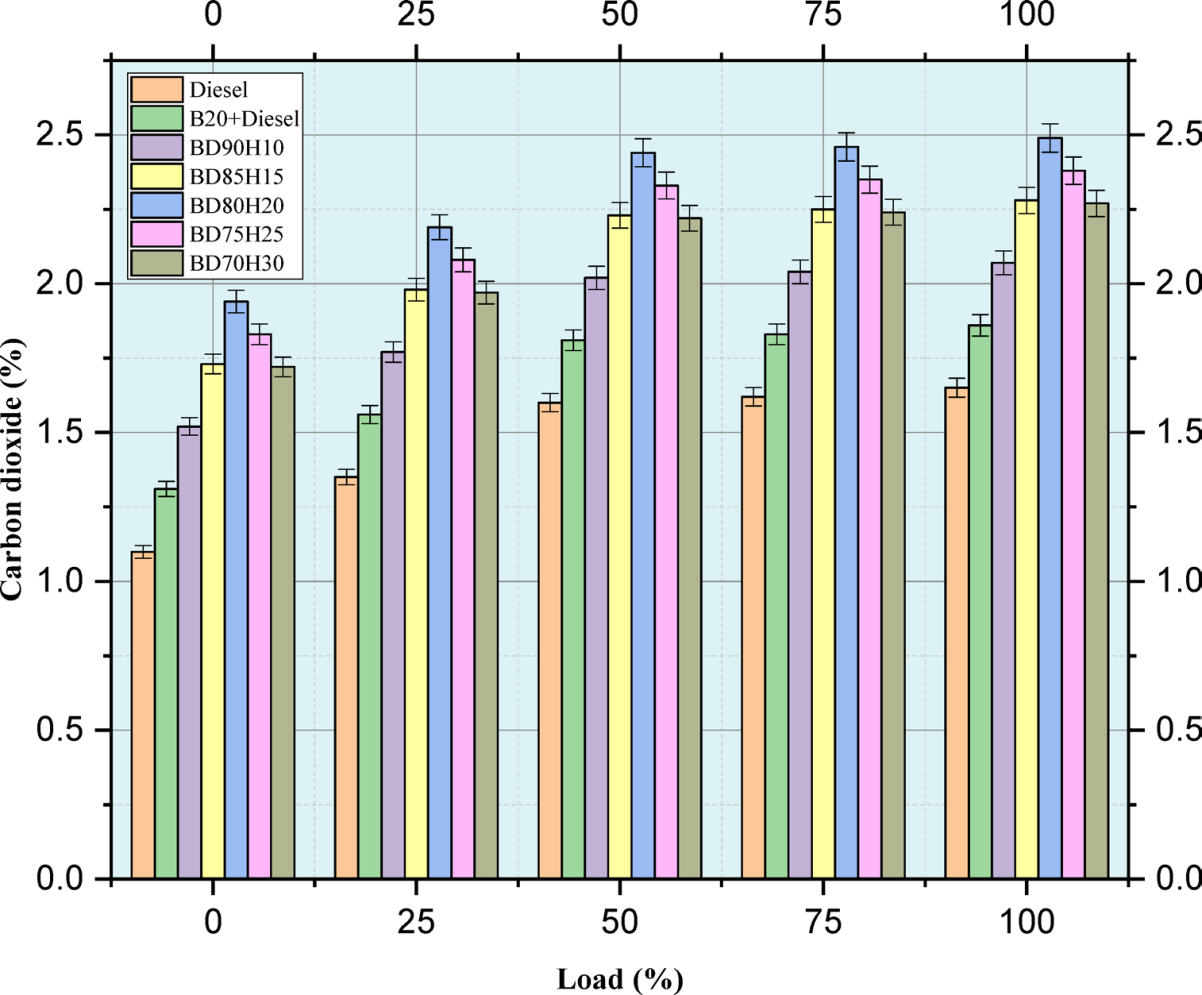
Load versus CO_2_ emission in %.

Figure 9 indicates the emission value of CO_2_ in percentage. Carbon Dioxide (CO_2_) emissions, one of the major greenhouse gases, were evaluated across different engine loads for a range of fuel blends from 0 to 100%. Lower CO_2_ emissions will be desirable in trying to counteract climate change.The test fuels included standard diesel, a 20% biodiesel and 80% diesel blend (B20), and several biodiesel-hydrogen combinations: BD90H10, BD85H15, BD80H20, BD75H25, and BD70H30. CO_2_ emissions tend to increase with engine load because more fuel is burned to produce higher power outputs. Pure diesel is the baseline^20^. The B20 blend has slightly higher CO_2_ emissions than pure diesel at all loads, which is consistent with the slightly lower energy content of biodiesel requiring more fuel to achieve the same power output. Introducing hydrogen into the biodiesel blend causes an additional rise in CO_2_ emissions^47^. The hydrogen blends exhibit increasingly higher CO_2_ emissions with increasing hydrogen concentration at all loads. This is probably because, though hydrogen contains no carbon itself, its blending can improve the combustion of the base fuel, and it thus results in a more complete oxidation of carbon in the biodiesel and diesel components. This implies that while hydrogen blends may have improvements in other emissions such as CO and HC, they are likely to incur increased CO_2_ emissions. More research and lifecycle analysis are necessary to fully evaluate the environmental impacts of these fuel blends, with regard to trade-offs between different emission species^43^.

#### Smoke

**Fig. 10:**
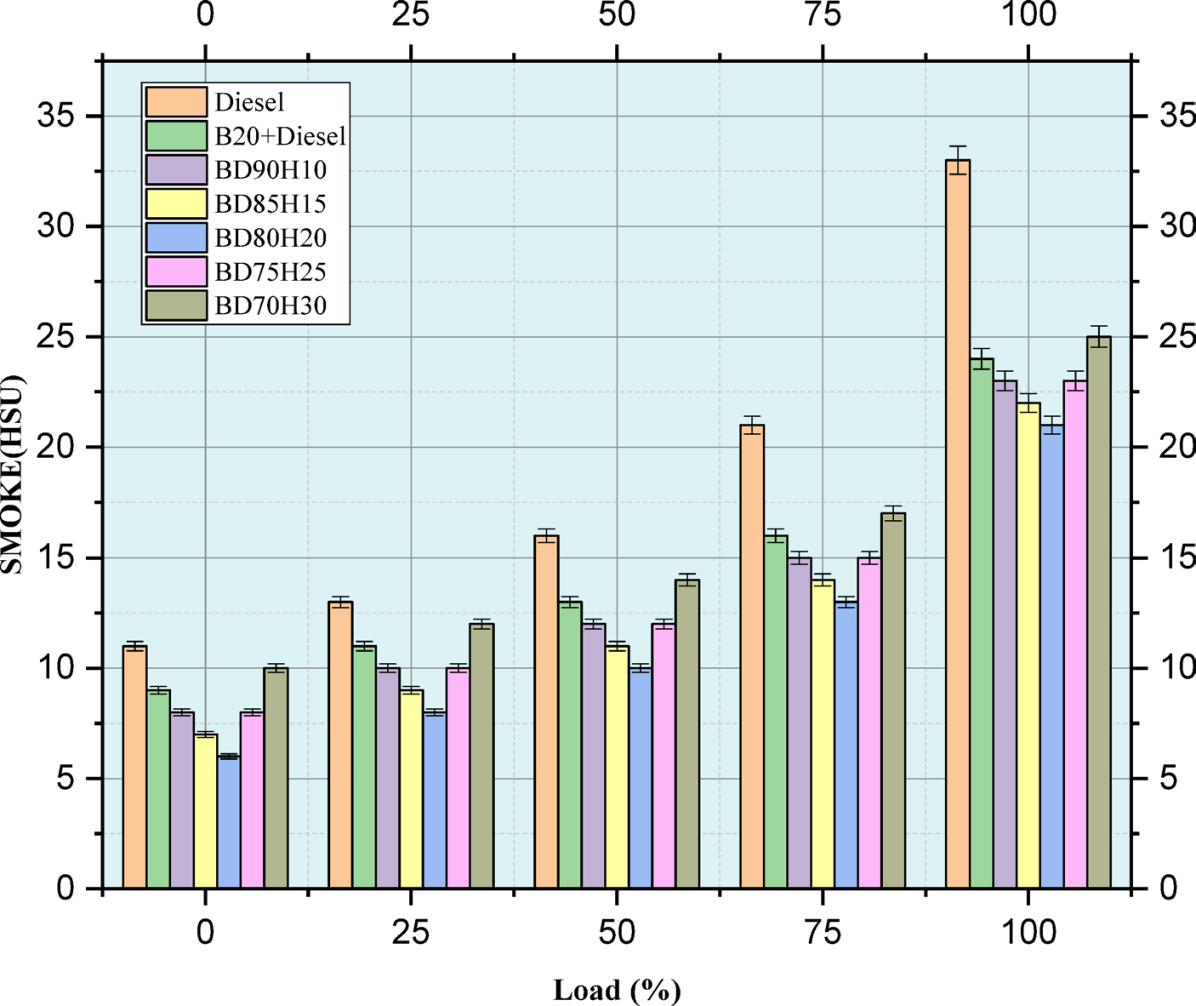
Load versus SMOKE emission in HSU.

Figure 10 indicates the emission value of SMOKE in HSU. Smoke opacity, measured in Hartridge Smoke Units (HSU), is measured over a range of engine loads (0–100%) for a number of fuel blends. Lower HSU values correspond to less smoke and hence better combustion. Standard diesel, a 20% biodiesel and 80% diesel blend (B20), and a number of biodiesel-hydrogen blends (BD90H10, BD85H15, BD80H20, BD75H25, and BD70H30) are evaluated. Smoke opacity tends to increase with increasing engine load since more fuel is burned. Pure diesel is the reference, and as expected, it has the highest smoke opacity of all the fuels tested at all loads^5^. The B20 blend has much lower smoke opacity than diesel, probably because the oxygen in biodiesel causes more complete combustion. Hydrogen addition to the biodiesel blend reduces smoke opacity even further. All blends of hydrogen consistently show the lowest smoke opacity at all loads; the effect is even much more pronounced at higher loads^17,28^. This in itself indicates that the influence of hydrogen on combustion improves the oxidation process to an extent that removes particulate matter emission significantly. The data clearly indicates that adding hydrogen to biodiesel blends is an effective way of minimizing smoke emissions across the whole range of the engine operating modes^5^.

#### NOx

**Fig. 11:**
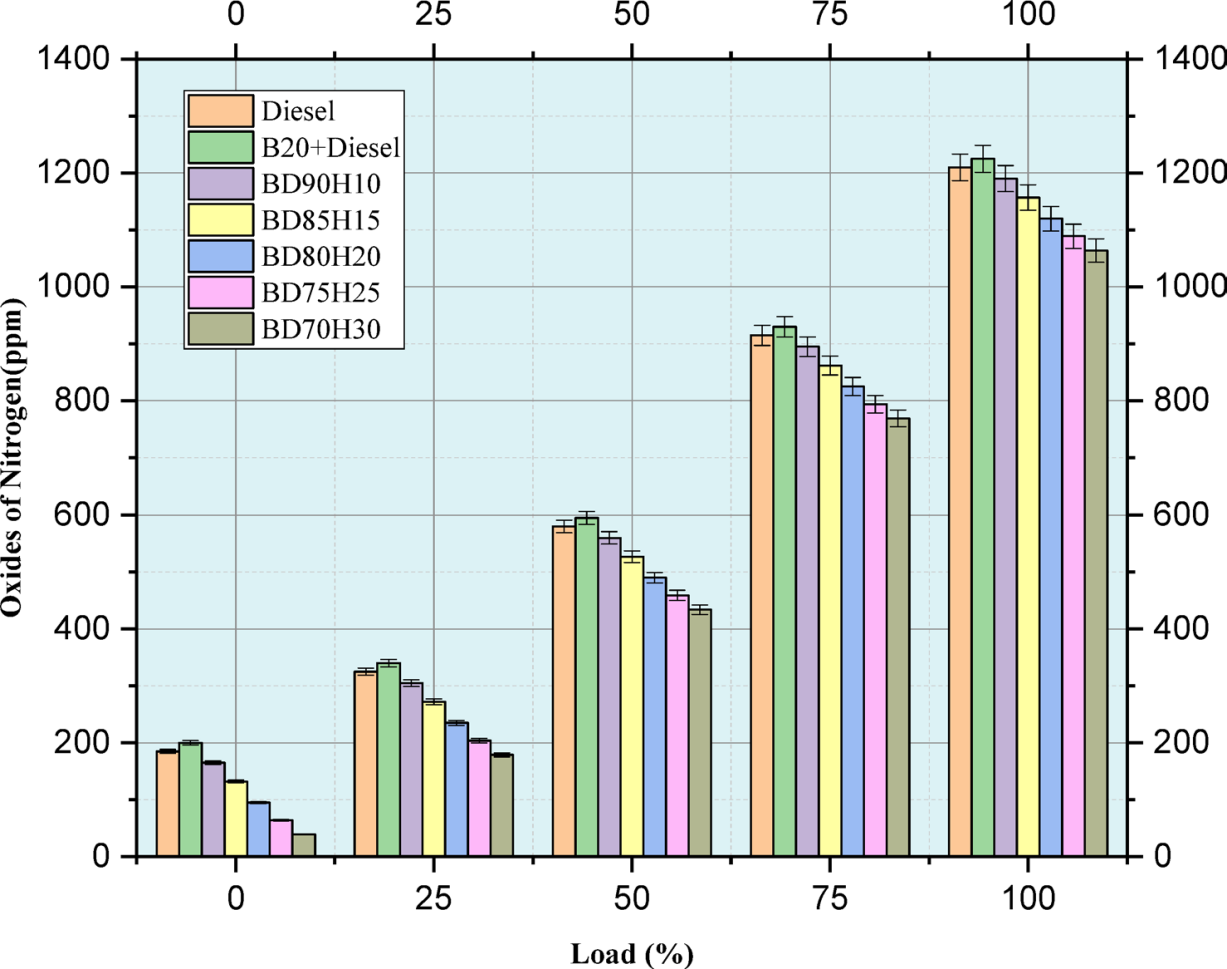
Load versus emission of oxides of nitrogen in ppm.

Figure 11 indicates the emission value of oxides of nitrogen. NOx is a significant air pollutant through releases from various engine loads (0–100%) using different fuel blends. NOx production is highly temperature-dependent and is often higher with increasing temperature. In this study, standard diesel, a 20% biodiesel and 80% diesel blend, B20, and several biodiesel-hydrogen combinations, BD90H10, BD85H15, BD80H20, BD75H25, and BD70H30, are compared. Generally, NOx emissions increase with engine load as combustion temperatures rise with increased power output. Pure diesel serves as the baseline. The B20 blend shows a small increase in NOx emissions above pure diesel for all loads. This could be due to slightly different combustion characteristics between biodiesel and diesel that can cause localized higher temperatures^25,48,49^. It was a complex task to introduce hydrogen into the biodiesel blend with regard to NOx emissions. At low loads, blends of hydrogen exhibited lower NOx emissions than that of diesel. This would be due to the water vapors from combustion, which decreases peak combustion temperatures. However, at higher load points, the hydrogen blends, especially with higher hydrogen fractions, tend to increase NOx emissions, reaching levels higher than both diesel and B20. This implies that although the presence of water may suppress NOx formation at lower temperatures, the overall increase in combustion efficiency and potentially higher peak temperatures due to hydrogen’s rapid combustion kinetics eventually becomes the dominant factor, leading to increased NOx production. The data emphasizes a complex interplay between load, hydrogen fraction, water formation, and NOx emissions, and careful optimization will be required to minimize NOx across the engine’s operating range^39^. Figure 10 shows that crank angle in deg. versus In-cylinder pressure rise in bar.

### Combustion parameters

#### In cylinder pressure

**Fig. 12:**
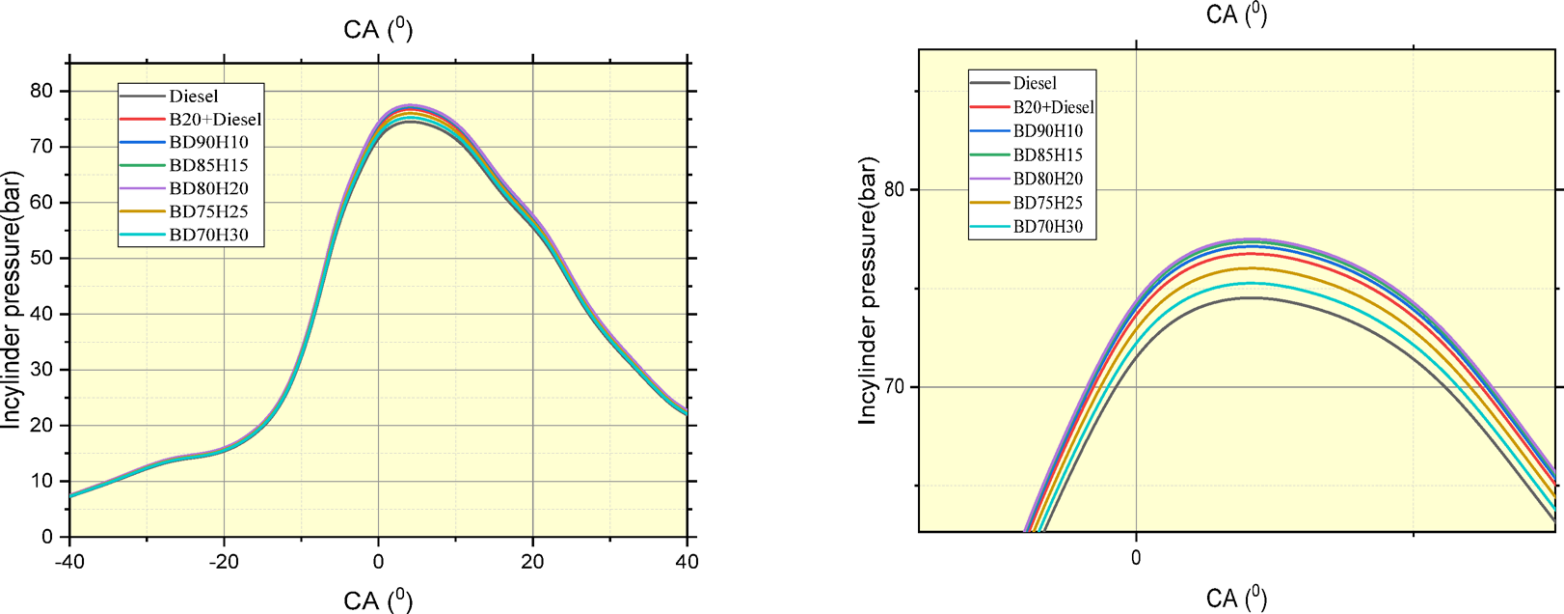
Crank angle in deg. Versus In-cylinder pressure rise in bar.

Figure 12 shows that Crank angle in deg. versus In-cylinder pressure rise in bar. In-cylinder pressure is a vital parameter showing the combustive process of an internal combustion engine and is evaluated at different crank angles for various fuel blends. The table presents the values of pressure (in bars or its equivalent pressure unit) in standard diesel, Biodiesel 20% blended with diesel 80% (B20), and some biodiesel-hydrogen mixtures (BD90H10, BD85H15, BD80H20, BD75H25, and BD70H30).The pressure rises from the piston as it moves during the compression stroke to near top dead center (TDC) where combustion is thought to take place, makes the analysis of combustion characteristics for each of these fuels possible based on the pressure curves. For example, differences in peak pressure and crank angle at that time may indicate dissimilar combustion timings and even heat-release rates. The rate of combustion is reflected by the slope of the pressure rise. Comparing diesel to B20, the effect of adding biodiesel can be seen in the pressure trace. In a similar fashion, analyzing the trends in hydrogen-blended fuels will elucidate how varying the hydrogen fraction affects in-cylinder pressure development. For instance, hydrogen blending could shift combustion phasing so that pressure increase is more rapid or slow relative to diesel or B20. The peak pressure at a given crank angle also provides information on the burn progression^49^.

#### Net heat release rate

**Fig. 13:**
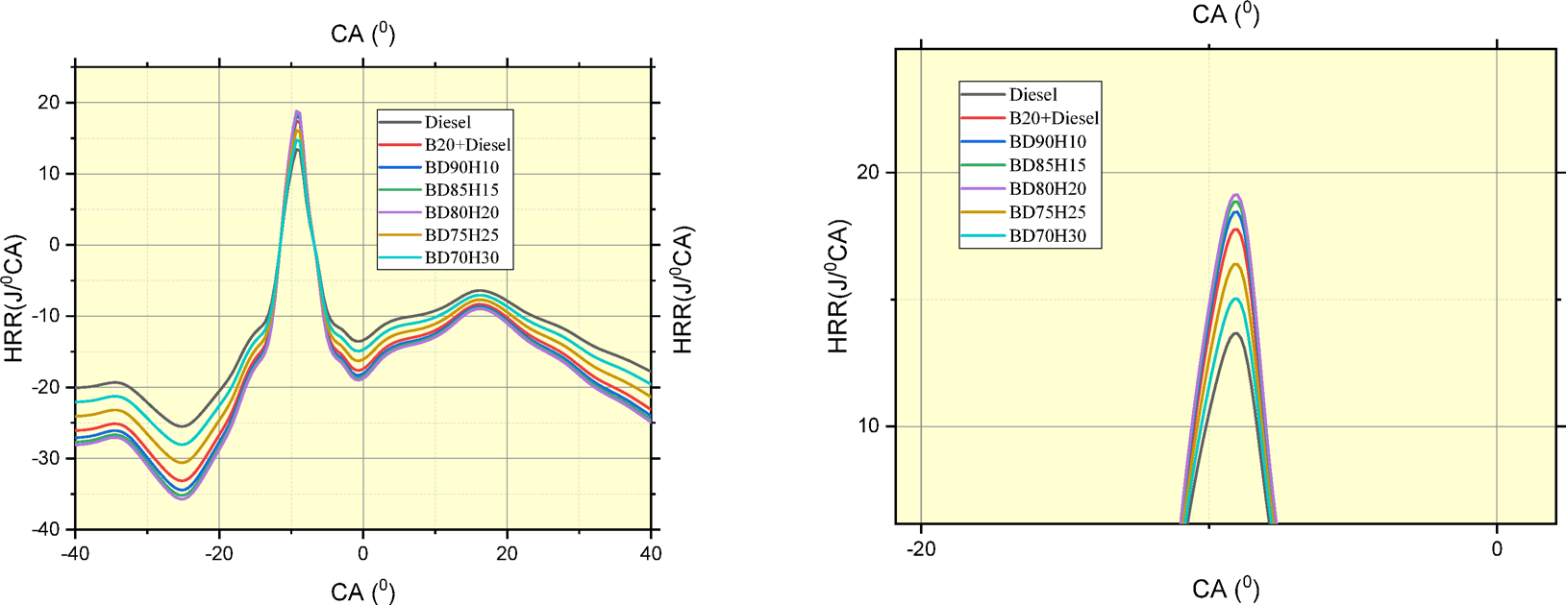
crank angle in deg. Versus Net heat release rate in j/^0^CA.

Figure 13 shows in-cylinder pressure traces of various fuel blends at crank angles, thus elucidating their combustion behavior. The tested fuels are standard diesel, a 20% biodiesel and 80% diesel blend (B20), and various biodiesel-hydrogen blends: BD90H10, BD85H15, BD80H20, BD75H25, and BD70H30. As observed from the data, they reflect the pressure variation within the cylinder when the piston is moving and combustion happens in the cylinder. This would imply changes in peak pressure values and the angle at which that peak pressure occurs, suggesting variability in combustion timing and rate of heat release. B20 has slightly lower peak pressures than diesel, likely because of the slightly lower energy content per unit mass of biodiesel compared to diesel fuel. This difference in energy content requires a slightly different combustion process to achieve similar power output, which manifests as a lower peak pressure^50^.

Across the hydrogen blends, there is a trend in peak pressure and its location that can be observed, indicating the effect of the change in hydrogen fractions on in-cylinder pressure development. In general, adding hydrogen to the biodiesel blend resulted in higher peak pressures than diesel and B20. Hydrogen has a higher flame speed and wider flammability limits than both diesel and biodiesel. This results in quicker and complete combustion, with an increased rise of pressure to higher peak pressures. However, the magnitude depends upon the particular fraction of hydrogen present in the blend^51^.

The optimal hydrogen fraction to maximize peak pressure is something that depends on a complex interaction of factors. Higher hydrogen fractions typically tend to produce more rapid and efficient combustion, but excessive hydrogen may shift the phasing of the combustion and possibly result in additional heat losses or other inefficiencies. Consequently, there will be a point of diminishing returns at which additional increases in hydrogen fraction do not provide a proportionate increase in peak pressure or even decrease^52^.

### Sustainability assessment study

Sustainability assessment has become imperative towards comprehensively understanding the impacts of projects, products, or policies across environmental, social, and economic dimensions. The leading trend of pursuit of sustainable practices has led to the growing demand for effective tools of assessment toward more informed decisions. This paper analyses the use of the Pugh matrix as a valuable constituent of a sustainability assessment. The Pugh matrix is a comparative and structured methodology that helps one compare options on a pre-determined set of sustainability criteria. This tool systematically scores options relative to a baseline and provides the identification of preferred sustainable solutions and pinpoint areas for potential improvement. This methodology, therefore, will promote transparency while allowing stakeholders to visualize the inherent trade-offs associated with different choices, thus promoting a more holistic and objective assessment of sustainability^53^.

#### PUGH matrix

A Pugh matrix was created to rank how the different fuel blends compare relative to a datum baseline, the standard diesel. This assessment considered a variety of parameters that defined sustainability, broadly categorized into efficiency in terms of fuel (BTE and BSFC), air pollutants (HC, CO, Smoke, and NOx emissions), emissions in terms of greenhouse gases (CO_2_), economic factors like quality and consistency in fuel, fuel storage and handling facilities, and feedstock availability, and finally, resource factors such as cultivation practices, regional factors, and lifecycle perspective^34^.

A comparison for each defined parameter of every alternative fuel blend against the datum given for diesel. A qualitative score system was utilized: “>”, performed better than the blend; “the blend performed worse”, “S” and, which was identical with diesel. This comparative approach thus facilitated quite clear understanding of each blend’s relative performance across the different sustainability dimensions. Other alternative fuels considered included B20, a blend of 20% biodiesel and 80% diesel, as well as the following combinations of biodiesel-hydrogen: BD90H10, BD85H15, BD80H20, BD75H25, and BD70H30, through which one could independently analyze the effects of the biodiesel and hydrogen addition as well as their interaction^31^.

After the pairwise comparisons, the scores for each fuel blend were tallied to determine the net score of “+”s (positive assessments) and “−”s (negative assessments). The overall ranking of the fuel blends was determined by the sum of these totals along with assigned weightings that reflected the relative importance of each parameter. This ranking process identified the most promising sustainable options. The weightings played a central role in the final outcome because they reflected the priorities of the study concerning the various aspects of sustainability. Thus, the Pugh matrix enabled the structured and transparent comparison of the inherent complex trade-offs of an evaluation of different fuel blends’ sustainability^54^. Table 4 shows the developed PUGH matrix from the experimental observations. Table 5 shows the developed PUGH matrix from the experimental observations.

**Table 5:** Developed PUGH matrix from the experimental observations.

	Parameters	Weightage	Diesel (Datum)	B20 + Diesel	BD90H10	BD85H15	BD80H20	BD75H25	BD70H30
Fuel efficiency	BTE (%)	2	− 2	− 1	1	1	1	1	1
	BSFC (kg/kWh)	2	− 2	− 1	1	1	1	1	1
Air pollutants	HC	2	− 1	− 1	1	1	1	1	1
	CO	2	− 1	− 1	1	1	1	1	1
	SMOKE	2	− 1	− 1	1	1	1	1	1
	NOX	2	− 1	− 1	1	1	2	2	2
Greenhouse gas emissions	CO_2_	2	2	− 1	− 1	− 1	− 1	− 2	− 2
Economic factors	Fuel quality and consistency	2	2	1	1	1	1	1	1
	Fuel storage and handling	2	2	1	− 2	− 2	− 2	− 2	− 2
Resource availability	Feedstock availability	2	− 2	1	1	1	1	1	1
	Cultivation practices	2	− 2	1	1	1	1	1	1
	Regional factors	2	2	2	1	1	1	1	1
	Lifecycle perspective	2	− 2	2	1	1	1	1	1
	Total	26	− 6	1	8	8	9	8	8
	Total (+) ives	NA	4	8	10	11	12	12	12
	Total (−) ives	NA	− 10	− 8	− 2	− 3	− 3	− 4	− 4
	Ranking	NA	4	3	2	2	1	2	2

#### Weightages provided

##### Fuel efficiency

Fuel efficiency is provided a weightage of 2 W and when considering fuel efficiency Brake thermal efficiency, and brake specific fuel consumption plays a vital role and hence when considering brake thermal efficiency diesel, B20D80, BD90H10, BD85H15, BD80H20, BD75H25, and BD70H30 has been provide score of − 2, − 1, 1, 1, 1, 1, & 1 for brake thermal efficiency and − 2, − 1, 1, 1, 1, 1, & 1 for brake specific fuel consumption respectively based on the observation − 1, − 1, 1, 1,1, 1, & 1 consecutively^34^.

##### Air pollutants

Air pollutants consist of HC, CO, Smoke and oxides of nitrogen, when considering the test fuels diesel, B20D80, BD90H10, BD85H15, BD80H20, BD75H25, and BD70H30 a score of 2W is given and when HC is considered a score of − 1,− 1, 1, 1, 1, 1, & 1 is given for the fuels respectively. Similarly CO, SMOKE and oxides of nitrogen have been provided with scores of − 1, − 1, 1, 1, 1, 1, & 1 for CO, − 1, − 1, 1, 1, 1, 1, & 1 and for NOx as − 1, − 1, 1, 1, 2, 2, & 2 for the test fuels consecutively^34^.

##### Greenhouse gas emission

Greenhouse gas from engine is CO_2_ and have been provided a weightage of 2 and the test fuels diesel, B20D80, BD90H10, BD85H15, BD80H20, BD75H25, and BD70H30 have been provided a score of 2, 2, − 1, − 1, − 1, − 1, − 2, & − 2 respectively^14^

##### Economic factors

Economic factors considered are fuel quality and consistency and fuel storage and handling, diesel and biofuel has no issues in quality and storage so score of 2 and 1 is given for diesel and B20D80 fuel but when considering hydrogen piloted fuel even though quality of fuel is good storage and handling take more precautionary measures in handling these hydrogen stored cylinder so BD90H10, BD85H15, BD80H20, BD75H25, and BD70H30 have been provided with 1, 1, 1, 1, 1, & 1 with fuel quality and consistency and for fuel handling and storage − 2, − 2, − 2, − 2, & − 2 have been provided for the fuels with hydrogen inline respectively^55^.

##### Resource availability

Resource available parameter is included with feed stock availability, cultivation practices, regional practices and life cycle perspective is considered, hence when discussing about diesel fuel, it’s a fossil fuel and is always in scarcity and feed stock availability, cultivation practices, regional practices and life cycle of diesel is given a score of − 2, 2, − 2, & − 2 respectively. But the biofuel *Andropogan narudus* is available all over south Asian region in all the water bodies and when considering hydrogen, it is the most abundant material all over the globe hence, the fuels B20D80, BD90H10, BD85H15, BD80H20, BD75H25, and BD70H30 have been provided scores for feed stock availability, cultivation practices, regional practices and life cycle perspective as 1, 1, 1, 1, 1, & 1 for feed stock availability 1, 1, 1, 1, 1, & 1 for cultivation process , 2,1,1, 1, 1, &, 1 for regional practices and for life cycle perspective a score of 2, 1, 1, 1, 1, & 1 is given for the fuels inline respectively^56^.

#### Identification of sustainable fuel

**Fig. 14:**
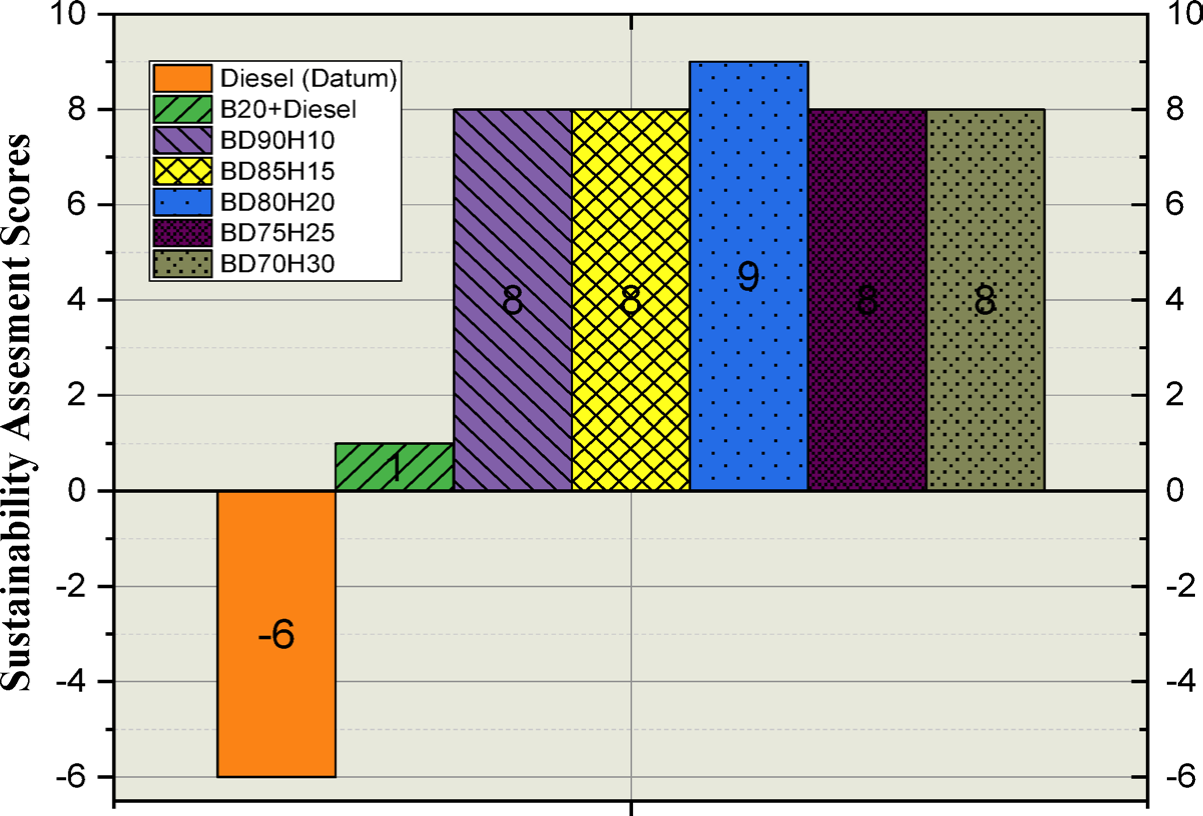
Sustainability assessment scores for all the test fuels.

From the development of PUGH matrix, the aggregated scores were calculated and provided rank for all the test fuels and Rank 1 is given to BD80H20 and Rank 2 is given for BD90H10, BD85H15, BD75H25, and BD70H30 and rank 3 is given for B20D80 fuel and Rank 4 is provided for Diesel fuel. So, from the statistical relationship BD80H20 Fuel is sustainability more balanced than all the test fuel conditions. Figure 14 indicates the aggregated scores of all the fuels^31^.

**Fig. 15:**
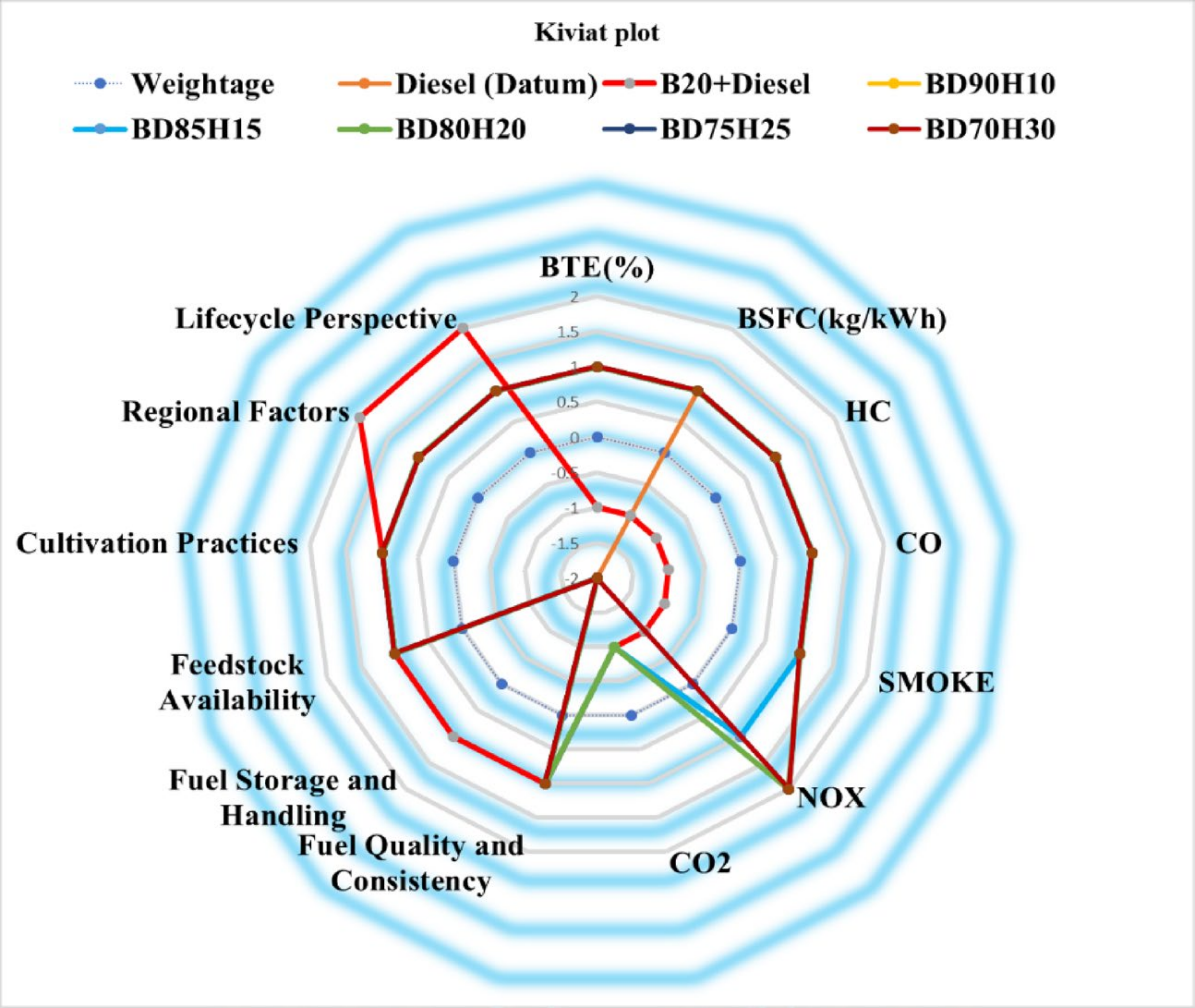
Kiviat plot for all the fuels and their corresponding Sustainable assessment scores.

Figure 15 shows the Kiviat plot for all the fuel and sustainability assessment scores. This chart is a graphical comparison of how sustainable different blends of fuel compared to a benchmark diesel fuel- the Datum. Every dimension on the plot represents a parameter, which includes the Brake Thermal Efficiency (BTE), Brake Specific Fuel Consumption (BSFC), emissions of HC, CO, Smoke, NOx, and CO_2_ besides economic and resource-related factors^23,57^. The relative performance of any fuel blend for a given parameter is indicated by the distance from the center along each axis. A positive value means better performance than diesel, and a negative value means poor performance. Lines are drawn to connect data points for each fuel blend in different colors, which then form polygons that represent the overall sustainability profile of the fuels. The plot makes it easy to compare the fuel blends, and trends and trade-offs across the various sustainability criteria can be easily seen. For example, one can see how the addition of biodiesel (B20) or varying proportions of hydrogen in biodiesel blends (BD90H10, etc.) affects performance relative to diesel across the range of parameters. The weightage assigned to each parameter is also visualized to show their relative importance in the overall sustainability assessment.

### ANN report

The developed Artificial Neural Network (ANN) model is designed to predict and validate complex, nonlinear experimental data for engine performance and emissions. Key parameters include Brake Thermal Efficiency (BTE), Brake Specific Energy Consumption (BSEC), hydrocarbons (HC), carbon monoxide (CO), carbon dioxide (CO_2_), nitrogen oxides (NOx), and smoke emissions. Implemented using MATLAB, the model processes engine load and fuel blend as input variables to forecast these metrics. For validation, 30 test patterns were utilized, with 70% of the data allocated for training the ANN model and the remaining 30% reserved for testing and validation.

**Fig. 16:**
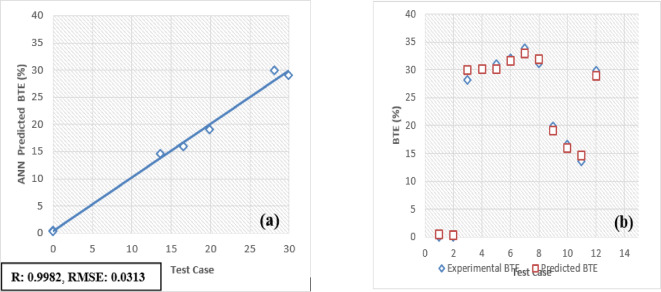
(**a**) BTE versus test case. (**b**) BTE versus expected and predicted BTE.

**Fig. 17:**
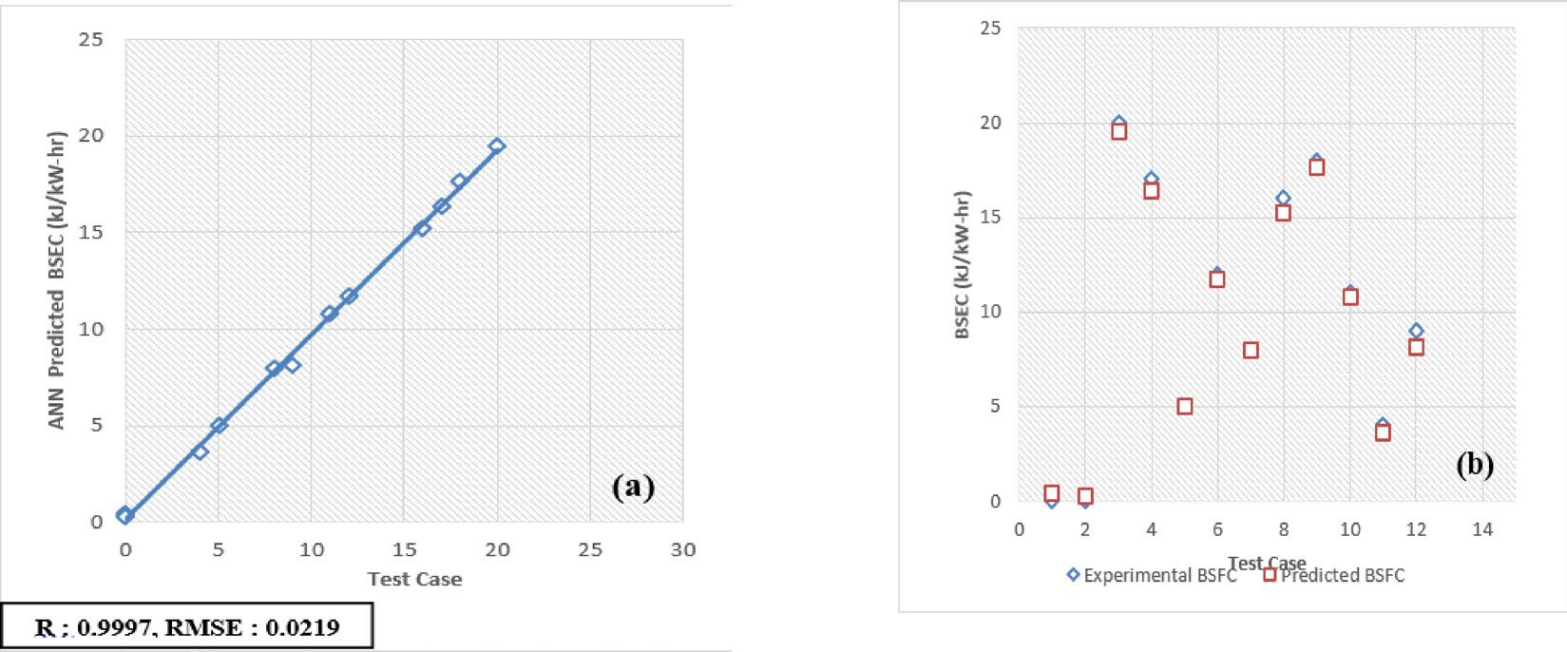
(**a**) BSFC versus test case. (**b**) BSFC versus expected and predicted BSFC.

**Fig. 18:**
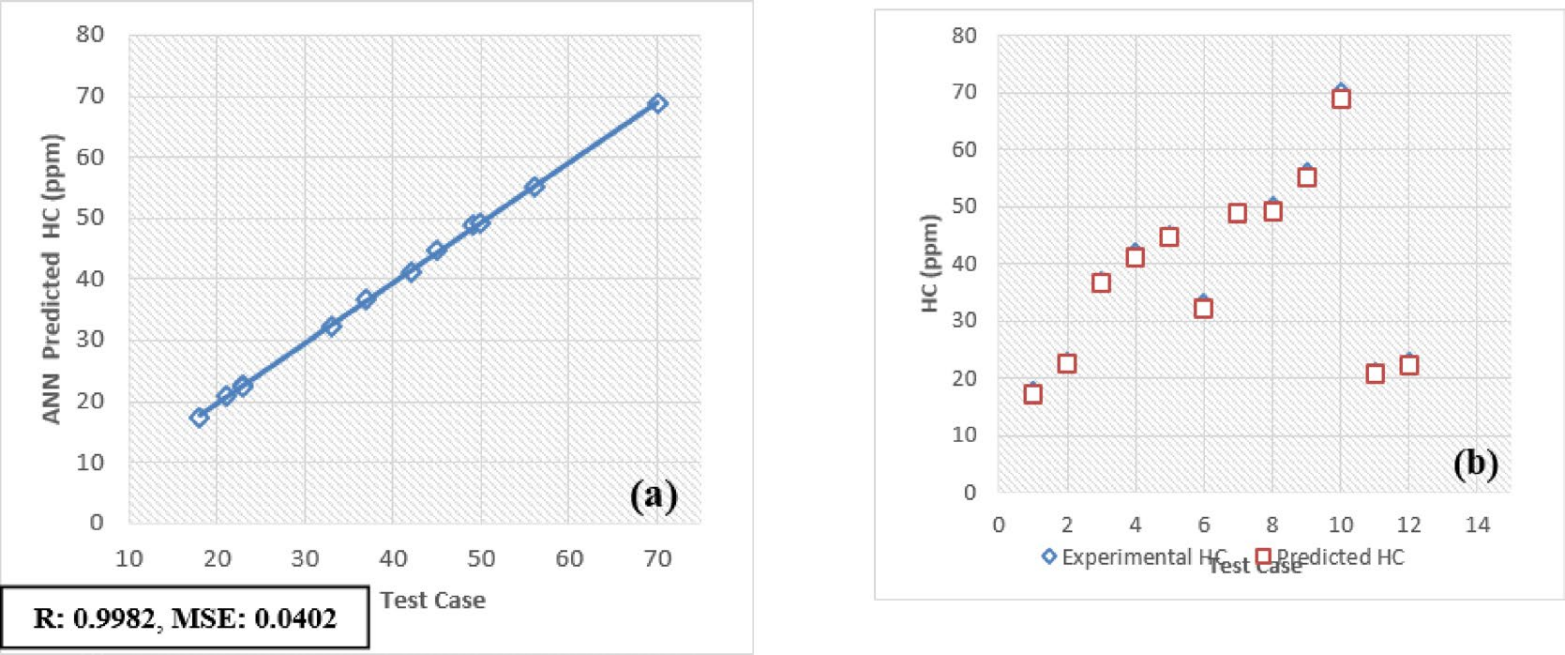
(**a**) HC versus Test Case. (**b**) HC versus expected and predicted HC.

**Fig. 19:**
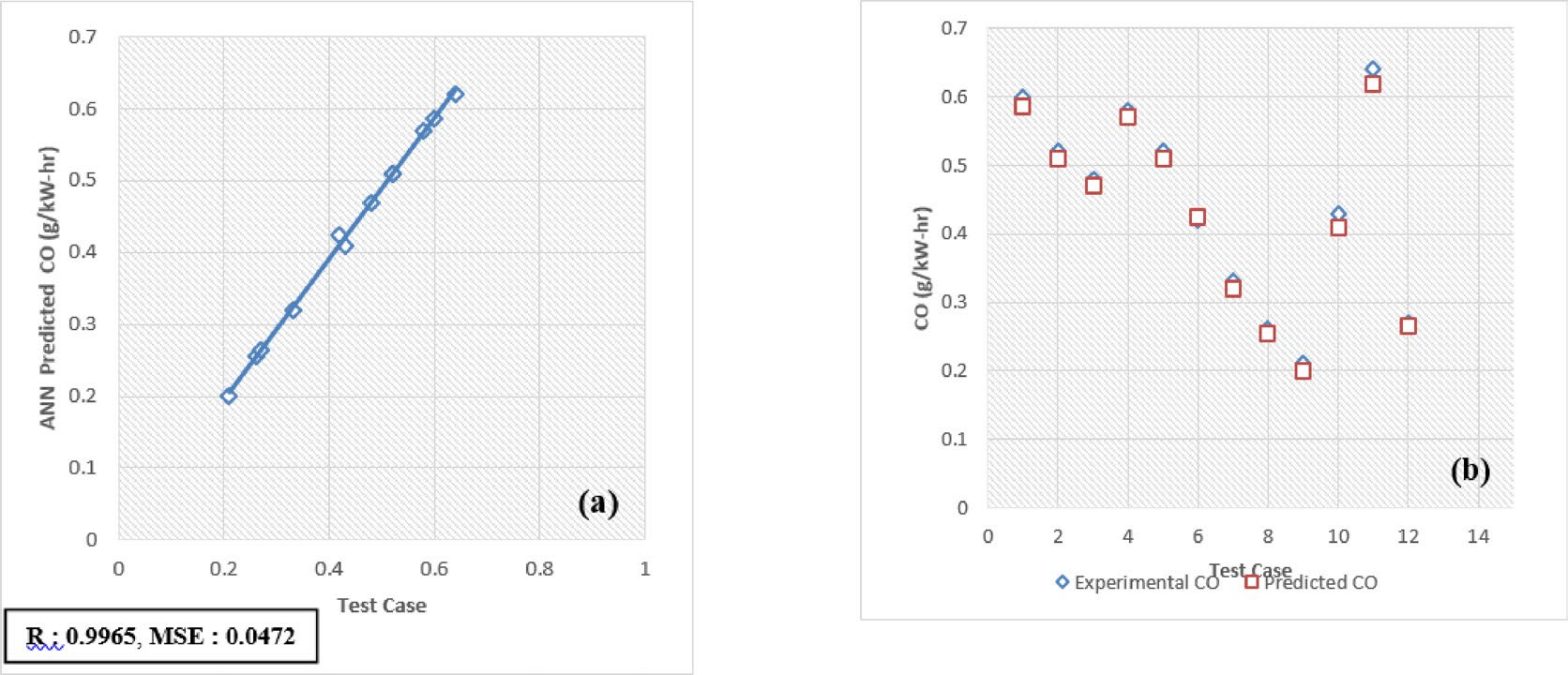
(**a**) CO versus Test Case. (**b**) CO versus expected and predicted CO.

**Fig. 20:**
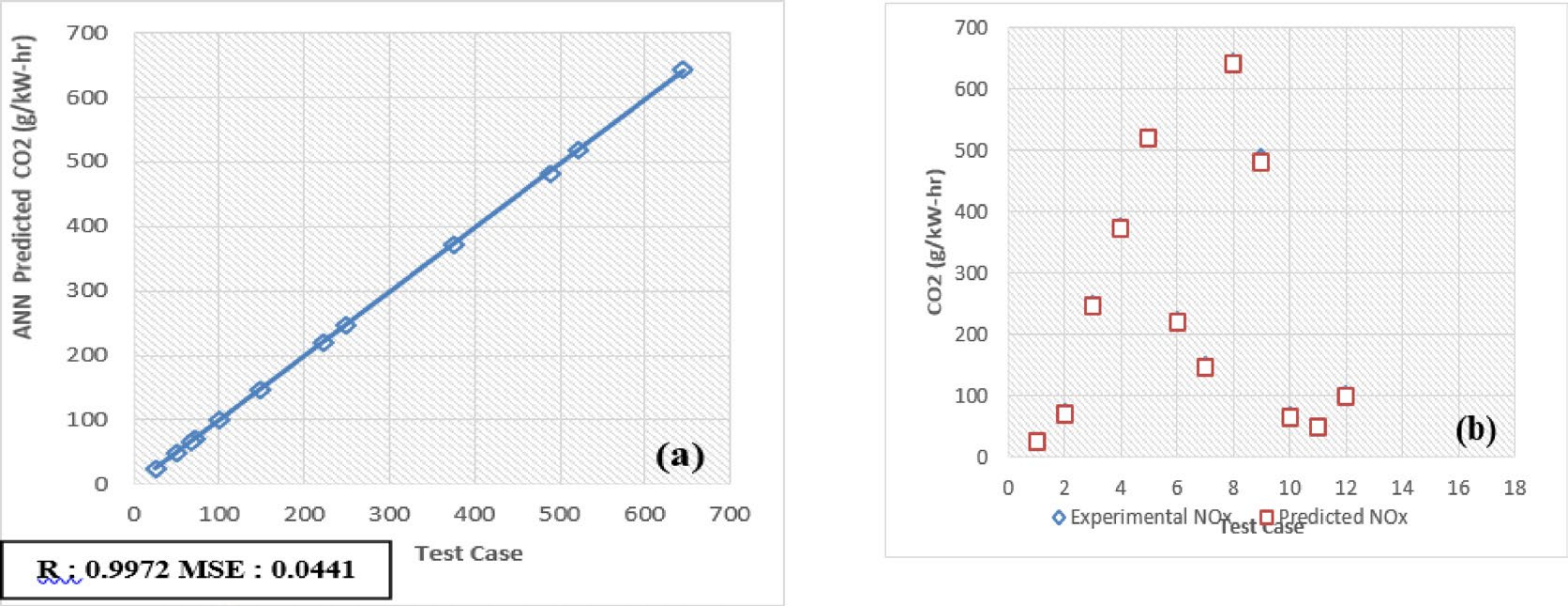
(**a**) CO_2_ versus Test Case. (**b**) CO_2_ versus expected and predicted CO_2_.

**Fig. 21:**
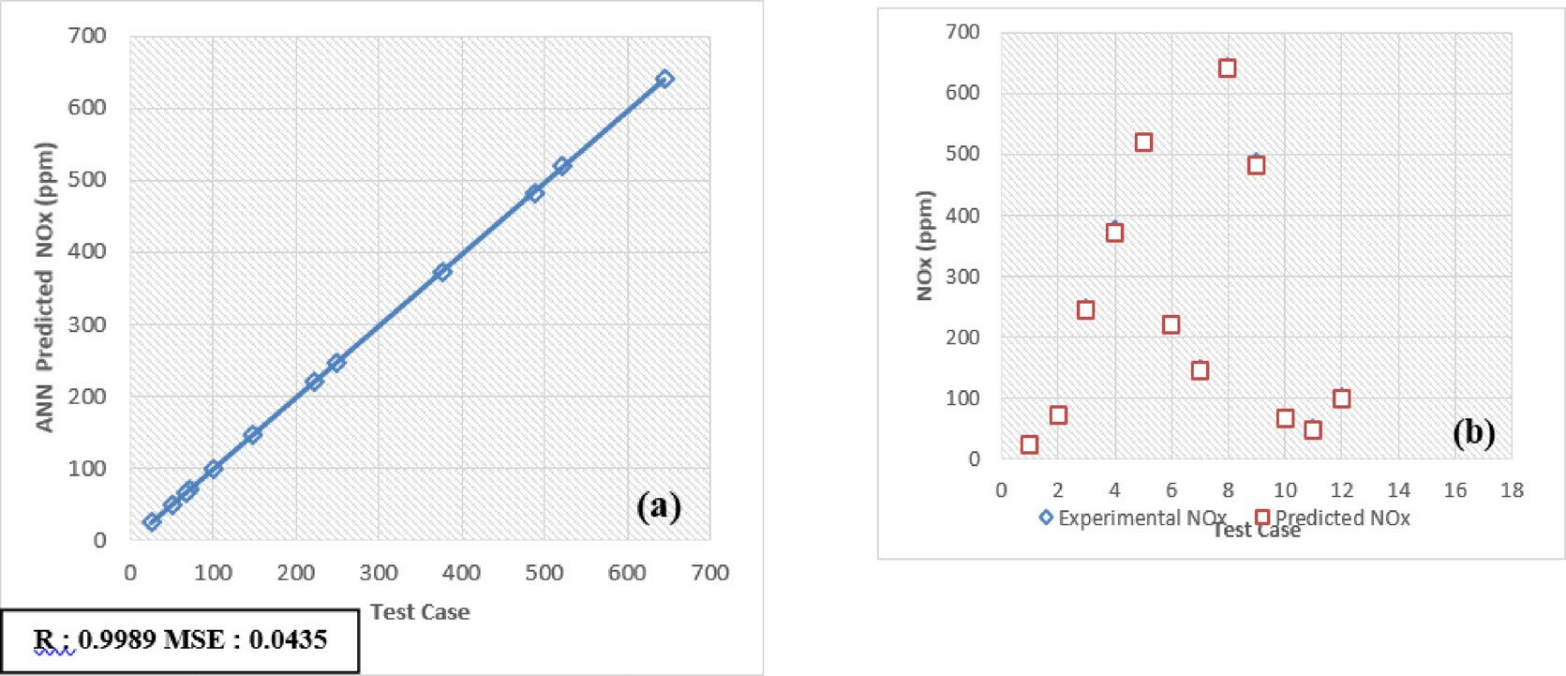
(**a**) NOx versus test case. (**b**) NOx versus expected and predicted NOx.

**Fig. 22:**
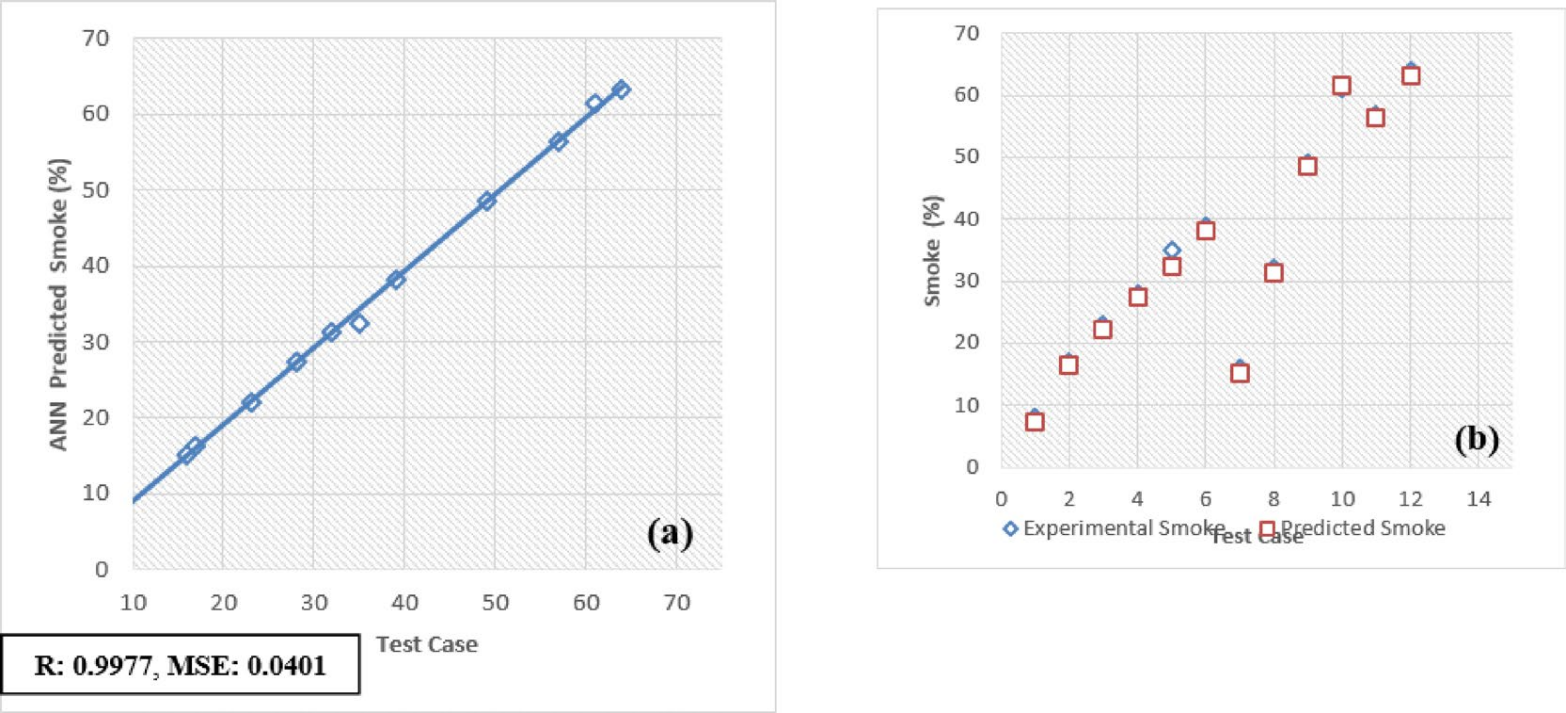
(**a**) Smoke versus test case. (**b**) Smoke versus expected and predicted smoke.

The selection of an optimal learning algorithm, based on the Regression coefficient (R), Mean Percentage Absolute Error (MPAE), and Mean Squared Error (MSE), was achieved through extensive trial and error to determine the appropriate algorithm and number of hidden neurons. Figures 16a, 17, 18, 19, 20, 21, and 22b illustrates a comparison between selected experimental results and ANN-simulated outputs. The R-values, ranging from 0.9965 to 0.9997, highlight a strong correlation for both performance and emission characteristics. Specific R-values for HC, CO, CO_2_, NOx, smoke, BTE, and BSEC are 0.9982, 0.9965, 0.9972, 0.9989, 0.9977, 0.9982, and 0.9997, respectively.

Similarly, Root Mean Square Error (RMSE) values span from 0.0217 to 0.0492, with respective RMSE values for BTE, BSEC, HC, CO, CO_2_, NOx, and smoke at 0.0313, 0.0219, 0.0402, 0.0472, 0.0441, 0.0435, and 0.040. Additionally, the MPAE values fall between 0.8 and 4%, with BTE, BSEC, HC, CO, CO_2_, NOx, and smoke reporting MPAE values of 1.2%, 0.8%, 4.2%, 3.4%, 1.69%, 1.98%, and 1.45%, respectively. These findings demonstrate that the developed ANN model maintains a relative error within 4%, which is within the acceptable range. Table 6 shows that predicated values.

**Table 6:** Predicated values.

Engine output	Selected algorithm	Number of neurons in hidden layers	R value	RMSE	MAPE (%)
BTE	SCG	11	0.9996	0.0313	1.21
BSEC	SCG	11	0.9991	0.0219	0.82
HC	LM	7	0.9982	0.0402	4.21
CO	RP	5	0.9965	0.0472	3.4
CO_2_	LM	7	0.9972	0.0441	1.69
Nox	LM	7	0.9989	0.0435	1.98
Smoke	BFGS	11	0.9977	0.0401	1.45

## Conclusion

This work shows that combining hydrogen in an RCCI engine with *Andropogon narudus* biodiesel results in performance, improved combustion quality, and significant environmental benefits.

- BD80H20 consistently produced the best results out of all the blends studied with a 3–5% improvement in Brake Thermal Efficiency, improved fuel economy by 5–8% on greater loads, and significantly lower HC, CO, and smoke emissions. Although the inclusion of hydrogen resulted in a significant increase in CO_2_ and NOx emissions (10–15%), better combustion and lower particulate matter emissions countered overall environmental impact.
- Furthermore, supporting better combustion dynamics in increase of enhanced combustion dynamics through higher flame velocity and hydrogen diffusivity was a rise in the in-cylinder pressure (5–10%).
- With respect to fuel efficiency, emissions, cost, and availability of resources, it was concluded in the Kiviat plot-based and Pugh matrix-based assessment that BD80H20 is the most sustainable blend.
- The study not only establishes the feasibility of machine learning application in real-time engine calibration but also confirms the reliability of Artificial Neural Networks (ANN) in model and prediction for direct use—achieving an RMSE of up to 0.9996 and an MPAE within an error margin of less than 4%.

Results indicate that biodiesel-hydrogen blends, particularly BD80H20, are potential alternatives to fulfill future energy and emission standards and minimize dependence on fossil fuels.

## Data Availability

The datasets generated during and/or analysed during the current study are available from the corresponding author on reasonable request.
